# The genome of *Eleocharis vivipara* elucidates the genetics of C_3_–C_4_ photosynthetic plasticity and karyotype evolution in the Cyperaceae

**DOI:** 10.1111/jipb.13765

**Published:** 2024-08-23

**Authors:** Hongbing Liu, Hang Zhao, Yanwen Zhang, Xiuli Li, Yi Zuo, Zhen Wu, Kaining Jin, Wenfei Xian, Wenzheng Wang, Weidong Ning, Zijian Liu, Xiaoxiao Zhao, Lei Wang, Rowan F. Sage, Tiegang Lu, Matt Stata, Shifeng Cheng

**Affiliations:** ^1^ Shenzhen Branch, Guangdong Laboratory of Lingnan Modern Agriculture, Genome Analysis Laboratory of the Ministry of Agriculture and Rural Affairs, Agricultural Genomics Institute at Shenzhen Chinese Academy of Agricultural Sciences Shenzhen 518120 China; ^2^ Biotechnology Research Institute Chinese Academy of Agricultural Sciences Beijing 100081 China; ^3^ Gembloux Agro‐Bio Tech, TERRA Teaching and Research Centre University of Liège Gembloux 4000 Belgium; ^4^ State Key Laboratory of Crop Stress Adaptation and Improvement, School of Life Sciences Henan University Kaifeng 475004 China; ^5^ Shenzhen Research Institute of Henan university Shenzhen 518000 China; ^6^ Key Laboratory of Plant Molecular Physiology, Institute of Botany China National Botanical Garden, Chinese Academy of Science Beijing 100093 China; ^7^ Department of Plant Sciences, Centre for Crop Systems Analysis Wageningen University & Research Wageningen 6708 WB The Netherlands; ^8^ Department of Ecology and Evolutionary Biology The University of Toronto Toronto M5S 3B2 ON Canada; ^9^ Plant Resilience Institute Michigan State University East Lansing 48824 MI USA; ^10^ Department of Biochemistry and Molecular Biology Michigan State University East Lansing 48824 MI USA

**Keywords:** C_4_ photosynthesis, Cyperaceae, *Eleocharis vivipara*, evolution

## Abstract

*Eleocharis vivipara*, an amphibious sedge in the Cyperaceae family, has several remarkable properties, most notably its alternate use of C_3_ photosynthesis underwater and C_4_ photosynthesis on land. However, the absence of genomic data has hindered its utility for evolutionary and genetic research. Here, we present a high‐quality genome for *E. vivipara*, representing the first chromosome‐level genome for the *Eleocharis* genus, with an approximate size of 965.22 Mb mainly distributed across 10 chromosomes. Its Hi–C pattern, chromosome clustering results, and one‐to‐one genome synteny across two subgroups indicates a tetraploid structure with chromosome count 2*n* = 4*x* = 20. Phylogenetic analysis suggests that *E. vivipara* diverged from *Cyperus esculentus* approximately 32.96 million years ago (Mya), and underwent a whole‐genome duplication (WGD) about 3.5 Mya. Numerous fusion and fission events were identified between the chromosomes of *E. vivipara* and its close relatives. We demonstrate that *E. vivipara* has holocentromeres, a chromosomal feature which can maintain the stability of such chromosomal rearrangements. Experimental transplantation and cross‐section studies showed its terrestrial culms developed C_4_ Kranz anatomy with increased number of chloroplasts in the bundle sheath (BS) cells. Gene expression and weighted gene co‐expression network analysis (WGCNA) showed overall elevated expression of core genes associated with the C_4_ pathway, and significant enrichment of genes related to modified culm anatomy and photosynthesis efficiency. We found evidence of mixed nicotinamide adenine dinucleotide ‐ malic enzyme and phosphoenolpyruvate carboxykinase type C_4_ photosynthesis in *E. vivipara*, and hypothesize that the evolution of C_4_ photosynthesis predates the WGD event. The mixed type is dominated by subgenome A and supplemented by subgenome B. Collectively, our findings not only shed light on the evolution of *E. vivipara* and karyotype within the Cyperaceae family, but also provide valuable insights into the transition between C_3_ and C_4_ photosynthesis, offering promising avenues for crop improvement and breeding.

## Graphical abstract

*Eleocharis vivipara* provides a prime model for studying photosynthetic plasticity, as it uses C_3_ photosynthesis underwater and C_4_ photosynthesis on land. The assembled genome and dynamic gene expression patterns provide new insights into the genetic basis of this photosynthetic transition, which can contribute to crop improvement and breeding strategies.

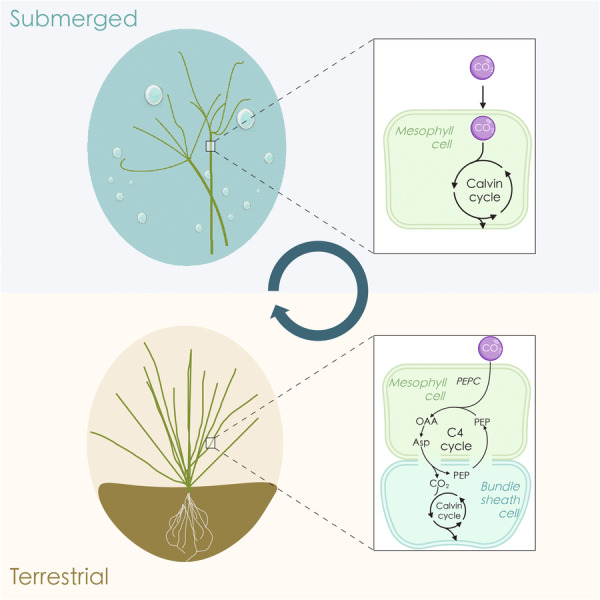

## INTRODUCTION

**Figure 1:**
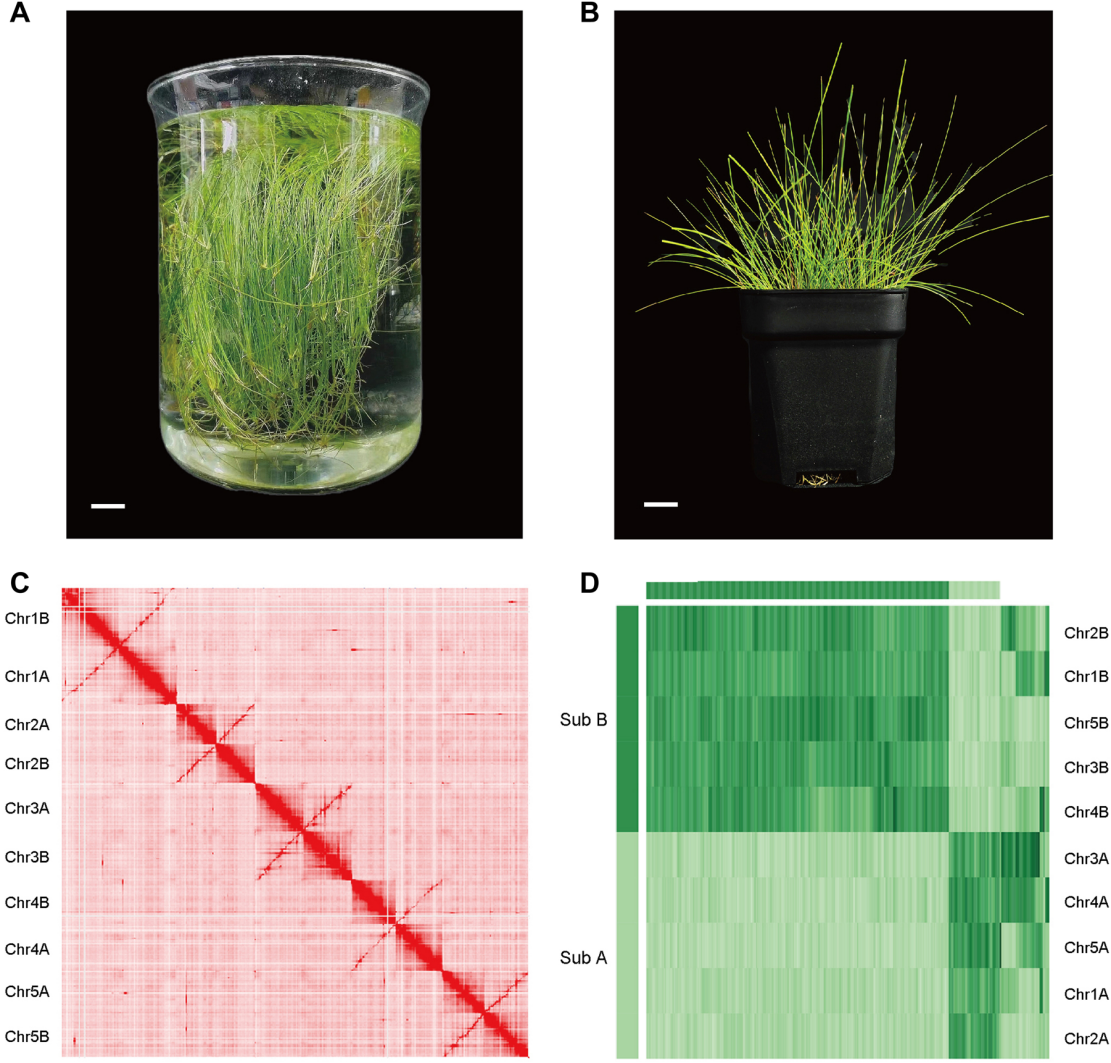
The phenotype of *Eleocharis vivipara* plant and chromosome patterns of its genome **(A**–**B)** Phenotype of *E. vivipara* in submerged **(A)** and terrestrial **(B)** state. For A, scale bar = 2.0 cm. For B, scale bar = 1.5 cm. **(C)** The Hi–C map of the 10 assembled chromosomes (*n* = 10). Here the 10 chromosomes formed a pattern which has five pairs of strong interaction intensity indicated by the red lines. **(D)**. The cluster results demonstrating two subgroups of chromosomes in *E. vivipara* genome (*n* = 2*x* = 10).

The sedge species *Eleocharis vivipara* is highly regarded for its ornamental, ecological, and genetic research significance (Ueno, 2001; Baksh and Richards, 2006). This species exhibits distinct structural traits, such as its leafless habit with slender, linear, photosynthetic culms, setting it apart from other genera within the Cyperaceae and Poaceae families, and contributing to its ornamental appeal (Figure 1A). Ecologically, *E. vivipara*'s amphibious lifestyle, thriving both in aquatic and terrestrial environments (Figure 1A, B), offers valuable potential for genetic and physiological insights for research on flood tolerance, crop improvement, and breeding (Murphy et al., 2007). Physiologically, it is distinguished by its unique photosynthetic plasticity, employing C_3_ photosynthesis underwater and C_4_ photosynthesis on land, making it a prime model for studying these different photosynthetic pathways within a single individual (Ueno et al., 1988; Chen et al., 2011). Despite its significance, the molecular mechanisms and gene regulation underlying these traits remain largely unexplored due to the absence of high‐quality genomic data.

The Cyperaceae family is the third largest in the monocots, and includes approximately 104 genera and around 5,400 species with a global distribution (Simpson et al., 2003; Roalson, 2008). The five largest genera are *Carex*, *Cyperus*, *Eleocharis*, *Rhynchospora*, and *Fimbristylis*, with approximately 2,000, 650, 250, 250, and 200 species, respectively. Many species within these genera have adapted to new and extreme habitats, demonstrating remarkable evolutionary potential (Murphy et al., 2007; Roalson, 2008; Can et al., 2020). Recent studies have generated high‐quality, chromosome‐level genomes of species from the *Carex*, *Cyperus*, and *Rhynchospora* genera, close relatives of *E. vivipara*, revealing significant chromosomal variations ranging from two to 54 chromosomes (Can et al., 2020; Dávid et al., 2021; Hofstatter et al., 2022; Zhao et al., 2023) (Table 1). Notably, *Rhynchospora tenuis* has only two chromosomes, the minimal number recorded in plants, while *Rhynchospora pubera*, presumed to be a diploid species, exhibits a complex genomic structure that hides unexpected octoploidy (Hofstatter et al., 2022). However, in comparison, *E. vivipara*'s genome size, basic chromosome number, ploidy level, and precise divergence time with its close relatives remain undefined.

**Table 1:** Summary of features for the assembled genomes in Cyperaceae and Juncaceae families

No.	Family	Species	Genome size (Gb)	Chromosome number	Ploidy level	Gene number	Transfer RNA number	Repeat content	References
1	Cyperaceae	*Carex cristatella*	0.30	35	2	26,500	404	40.04	(Planta et al., 2022)
2		*Carex scoparia*	0.30	31	2	25,799	402	39.93	(Planta et al., 2022)
3		*Carex parvula*	0.78	32	4	45,002	1,495	52.47	(Qu et al., 2022)
4		*Carex kokanica*	0.67	Na	na	36,709	275	55.47	(Qu et al., 2022)
5		*Carex littledalei*	0.37	29	2	23,136	505	54.12	(Can et al., 2020; Ning et al., 2023)
6		*Carex myosuroides*	0.40	29	2	26,748	718	51.89	(Ning et al., 2023)
7		*Cyperus esculentus*	0.23	54	na	23,613	503	33.90	(Zhao et al., 2023)
8		*Rhynchospora pubera*	1.70	5	2	91,363	29,003	49.50	(Hofstatter et al., 2022)
9		*Rhynchospora breviuscula*	0.42	5	2	24,354	5,911	50.19	(Hofstatter et al., 2022)
10		*Rhynchospora tenuis*	0.40	2	2	23,215	4,029	45.40	(Hofstatter et al., 2022)
11		*Eleocharis vivipara* *	0.96	10	4	38,769	1,092	63.84	
12	Juncaceae	*Juncus effusus*	0.22	21	2	25,967	350	48.64	(Planta et al., 2022)
13		*Juncus inflexus*	0.27	21	2	25,422	372	42.73	(Planta et al., 2022)

The red asterisk denotes the *Eleocharis vivipara* genome assembly, na, not available.

While chromosome fusion and fission events are recognized as potential speciation mechanisms, their accumulation often leads to detrimental effects on individuals and populations, typically manifesting as problems with aneuploid gametes during meiosis or mitosis (Lukhtanov et al., 2018). In species with monocentromeric chromosomes, the mechanical forces during anaphase are exerted at a single point, causing the chromosome arms to trail behind and form the classic V‐shaped pattern. Such chromosomes are prone to abnormal or defective centromere activity following fusion or fission, leading to improper segregation. Conversely, species with holocentric chromosomes display parallel chromatid migration to the spindle poles during anaphase, maintaining normal centromere activity even in fused or fragmented chromatids, ensuring proper segregation (Wrensch et al., 1994; Mandrioli and Manicardi, 2020). Holocentric chromosomes thus contribute to chromosomal stability, more dynamic karyotypic evolution and promoting speciation (Monti et al., 2012; Manicardi et al., 2015; Lukhtanov et al., 2018). Additionally, the holocentromeres influence spatial genome organization, as evidenced by distinctive patterns in transposable element (TE) and gene densities, and Hi–C interaction intensities, compared to monocentric chromosomes (Hofstatter et al., 2022). In Cyperaceae, holocentromeres, monocentromeres, and heterogeneous centromeres were found in the species of *Rhynchospora, Scirpus* and *Bolboschoenus* genera, respectively (Nijalingappa, 1974; Melters et al., 2012; Hofstatter et al., 2022; Ning et al., 2024). However, patterns of chromosome rearrangements and the centromere organization in *E. vivipara* have yet to be elucidated.

Unlike maize and rice, which constitutively utilize either C_4_ or C_3_ photosynthesis, respectively, *E. vivipara* switches between C_3_ and C_4_ photosynthesis in aquatic versus terrestrial environments (Ueno et al., 1988; Chen et al., 2011). This variation manifests in significant anatomical, ultrastructural, and biochemical dissimilarity in different conditions, including differences in culm structures, the distribution and quantity of chloroplasts and mitochondria in bundle sheath (BS) and mesophyll (M) cells, enzyme activity, and metabolism (Ueno et al., 1988). C_4_ plants are generally classified into three metabolic subtypes according to their major decarboxylation enzyme nicotinamide adenine dinucleotide ‐ malic enzyme (NAD‐ME), NAD phosphate (NADP)‐ME (NADP‐ME), or phosphoenolpyruvate carboxykinase (PCK) (Maier et al., 2011; Wang et al., 2014). Although the terrestrial culms of *E. vivipara* were determined to utilize the NAD‐ME C_4_ subtype based on enzyme activity (Ueno et al., 1988), the specific genes involved in C_4_ pathway remains ambiguous due to lack of expression data based on a reference genome. Additionally, *Eleocharis baldwinii*, a closely related species, employs C_3_–C_4_ intermediate photosynthesis underwater and C_4_ type on land (Chen et al., 2014). The extent of gene expression changes accompanying this photosynthetic transition has been examined in both species, with a large disparity in results: 24,204 differentially expressed genes (DEGs) were associated with the aquatic to terrestrial transition in *E. baldwinii*, while only 56 were identified in *E. vivipara* (Chen et al., 2011; Chen et al., 2014). This disparity may be due to different methods, as the latter were identified through suppression subtractive hybridization library construction and array hybridization methods, which offer relatively low resolution (Liu et al., 2011; Külahoglu et al., 2014). A recent study has performed a *de novo* transcriptome analysis of submerged and terrestrial culms of *E. vivipara*, and identified the core C_4_ cycle genes (Harada et al., 2018). The research also conducted functional annotation and gene family expansion analysis, offering insight into the genetics of C_4_ evolution. However, the study did not include a comprehensive expression analysis to identify DEGs during photosynthesis type transition. Moreover, *de novo* transcriptome assemblies fail to account for the underlying genome structure, which will provide crucial context for the differential gene expression patterns associated with the establishment of C_4_ photosynthesis.

In this study, we present a high‐quality genome for *E. vivipara*, marking the first chromosome‐level genome within the *Eleocharis* genus, achieved through the integration of DNA Nanoball (DNB) short reads, PacBio, and Hi–C data. The resulting genome is 965.22 Mb in size, with a contig N50 of 2.17 Mb. Utilizing this high‐quality, chromosome‐scale genome, we are able to investigate this species' ploidy, accurately ascertain its phylogenetic position and divergence time from related clades, comparatively analyze chromosomal rearrangements between *E. vivipara* and related sedge species, and further investigate its centromere structure and 3D‐genome organization. Moreover, this genome enables a detailed dissection of the transition between C_3_ and C_4_ photosynthesis within a single genotype and elucidates specific genetic details of the C_4_ photosynthetic pathways employed in this species. The data generated and the analyses performed in this study will contribute to unraveling the karyotype evolution within the Cyperaceae family, a clade which has particularly dynamic and dramatic changes in chromosome organization (Roalson, 2008; Hipp et al., 2009), and lay a solid foundation for invaluable insights into the genetic control of C_3_ and C_4_ photosynthesis, offering substantial benefits for crop improvement and breeding initiatives.

## RESULTS

### *E. vivipara* is a tetraploid with 2*n* = 4*x* = 20 chromosomes

To assemble the chromosome‐level genome sequence for *E. vivipara*, we initially generated 88.25 Gb of short‐read data (Table S1). Using this data, we estimated its genome size is approximately 1.05 Gb with a heterozygosity ratio of about 0.65% (Figure S1A). Subsequently, we generated 77.83 Gb of continuous long‐read (CLR) data using PacBio, achieving around 74× coverage depth. Additionally, we sequenced 69.66 Gb of Hi–C data, with about 66× coverage. The PacBio long‐reads were assembled into 1,822 contigs, culminating in a genome size of 964.84 Mb and a contig N50 of roughly 2.17 Mb, utilizing Canu v2.0 (Table S2). Benchmarking Universal Single‐Copy Orthologs (BUSCO) evaluation against the embryophyta database indicated a genome completeness of approximately 94.23%. And 95.80% of the short‐read data was successfully mapped to the assembled genome (Figure S1B; Table S3), demonstrating its high continuity, integrity, and completeness. After improving contigs accuracy with short reads, we partitioned, clustered, and anchored 928.54 Mb of sequences into 10 chromosomes, leaving 36.68 Mb unclassified, through the application of Hi–C interaction data and 3D‐DNA software (Figure 1C). The sequence anchor ratio to the chromosomes was 96.20%, and chromosome length are between 77.72 and 116.72 Mb (Table S4). In summary, we produced a high‐quality, chromosome‐level genome for *E. vivipara*, measuring approximately 965.22 Mb, surpassing the size of most species within the Cyperaceae family, except for *R. pubera*, which is approximately 1.70 Gb and exhibits a hidden octoploidy level (Hofstatter et al., 2022) (Table 1).

The Hi–C interaction intensity pattern identified five highly similar pairs of chromosomes, with chromosomes in each pair exhibiting a high‐intensity diagonal characteristic (Figure 1C). These results are indicative of a potential tetraploid organization, aligning with recently published polyploid genomes (Zhang et al., 2018; VanBuren et al., 2020). Further analysis using SubPhaser demonstrated that the 10 chromosomes of *E. vivipara* could be categorized into two subgroups based on their 15‐mer sequences, here we label them as subgenome A and B (Figure 1D). These findings indicate that the sequenced *E. vivipara* genome is a tetraploid, with 2*n* = 4*x* = 20 chromosomes.

### Tetraploidy and repeat content contribute to *E. vivipara* larger genome size

**Figure 2:**
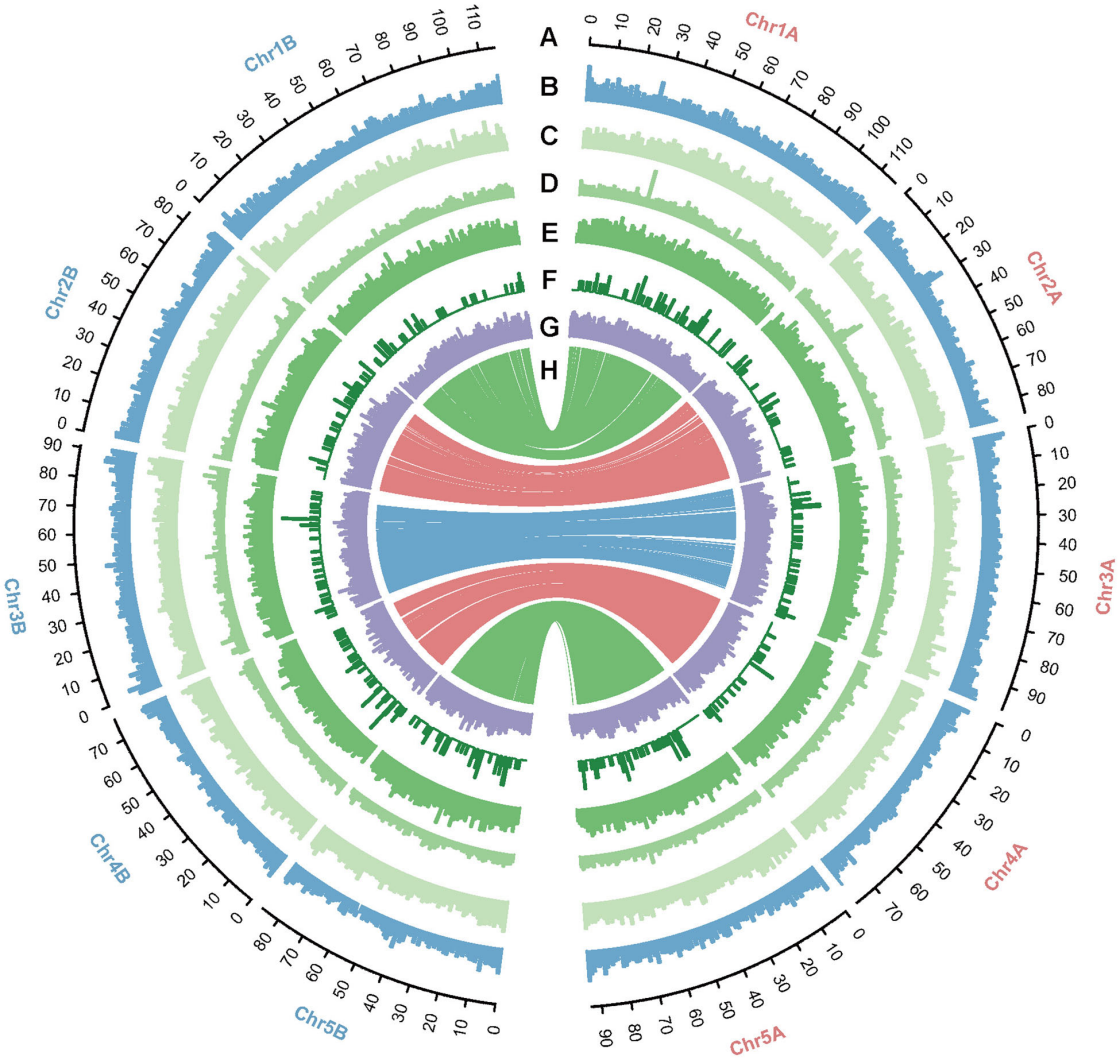
The genomic landscape of *Eleocharis vivipara* **(A)** Chromosome number and length (Mb). The chromosome names in subgenome A are labeled with red color; and in subgenome B, they are labeled with blue color. **(B)** GC (guanine/cytosine) content of the genome (~33% to 39%). **(C)** The *Copia* density of the genome (0 to 60 per Mb). **(D)**. The *Gypsy* density of the genome (0 to 100 per Mb). **(E)** The *Helitron* density of the genome (0 to 87 per Mb). **(F)** The satellite density of the genome (0 to 4 per Mb). **(G)** The gene density of the genome (0 to 75 per Mb). **(H)** Gene synteny between chromosome pairs across the two subgenomes.

Polyploidy and repeat content are well recognized as primary contributors to increased genome size in plants (Leitch and Leitch, 2012; Bennetzen and Wang, 2014), such as the recently published genome of *Panicum miliaceum* in the Poaceae family (Shi et al., 2019), *Vernicia fordii* and *V. montana* in Euphorbiaceae (Zhang et al., 2019; Li et al., 2024), and *Idesia polycarpa* in the Salicaceae family (Zuo et al., 2024). The extent of repeated sequences in *E. vivipara* was examined to assess its contribution to genome size. Utilizing Extensive de‐novo TE Annotator (EDTA), RepeatModeler, and RepeatMasker, we determined that repeat sequences constitute 63.84% of the *E. vivipara* genome (Tables 1, S5), surpassing the proportions found in closely related species within the Cyperaceae and Juncaceae families, which range from approximately 33.90% to 55.47% (Can et al., 2020; Hofstatter et al., 2022; Planta et al., 2022; Qu et al., 2022; Ning et al., 2023; Zhao et al., 2023). Consistent with the related Poaceae family, where long terminal repeat (LTR) retrotransposons predominate (Bennetzen, 2000; Bennetzen and Wang, 2014; Liu et al., 2020), the *E. vivipara* genome is mainly composed of LTR elements (26.65%), particularly *Copia* and *Gypsy*, and *Helitron* transposons (13.70%) (Figure 2; Table S5). These findings indicate that the expansion of LTR retrotransposons and *Helitron* transposons contribute substantially to *E. vivipara*'s genome size, second only to polyploidy.

We identified 38,769 protein‐coding and 1,092 transfer RNA (tRNA)‐coding genes in the *E. vivipara* genome using the Maker pipeline (Table 1). When compared to gene counts in other Cyperaceae and Juncaceae species, we found that the total number of its protein‐coding genes is consistent with tetraploid species: it is roughly double the gene count in diploid species (23,136–26,748), comparable to that of another tetraploid (45,002), and half that of the hidden octoploid (91,363) (Can et al., 2020; Hofstatter et al., 2022; Planta et al., 2022; Qu et al., 2022; Ning et al., 2023; Zhao et al., 2023). Protein‐coding genes occupy approximately 161.02 Mb of the genome (Figure 2), with average length of genes and transcripts being approximately 4,476 bp and 4,377 bp, respectively. The mean number of transcriptional isoforms per gene is 1.58 (Table S6). Functional annotation indicates that 90.52% of the genes (35,094 of 38,769) could be annotated, with the NR (Non‐Redundant Protein Sequence Database), Swissprot, Pfam (Protein families), KEGG (Kyoto Encyclopedia of Genes and Genomes), and GO (Gene Ontology) databases yielding annotations for 35,048, 29,825, 28,210, 17,135, and 17,011 genes, respectively (Figure S1C; Table S7). Comparative analysis based on the best hit for each gene in the NR database demonstrated that 79.14%, 4.66%, and 2.13% of genes in *E. vivipara* have a best match in *Carex littledalei*, *Ananas comosus*, and *Elaeis guineensis*, respectively (Figure S1D).

### *Eleocharis* has a closer relationship with the *Cyperus* genus in phylogeny

**Figure 3:**
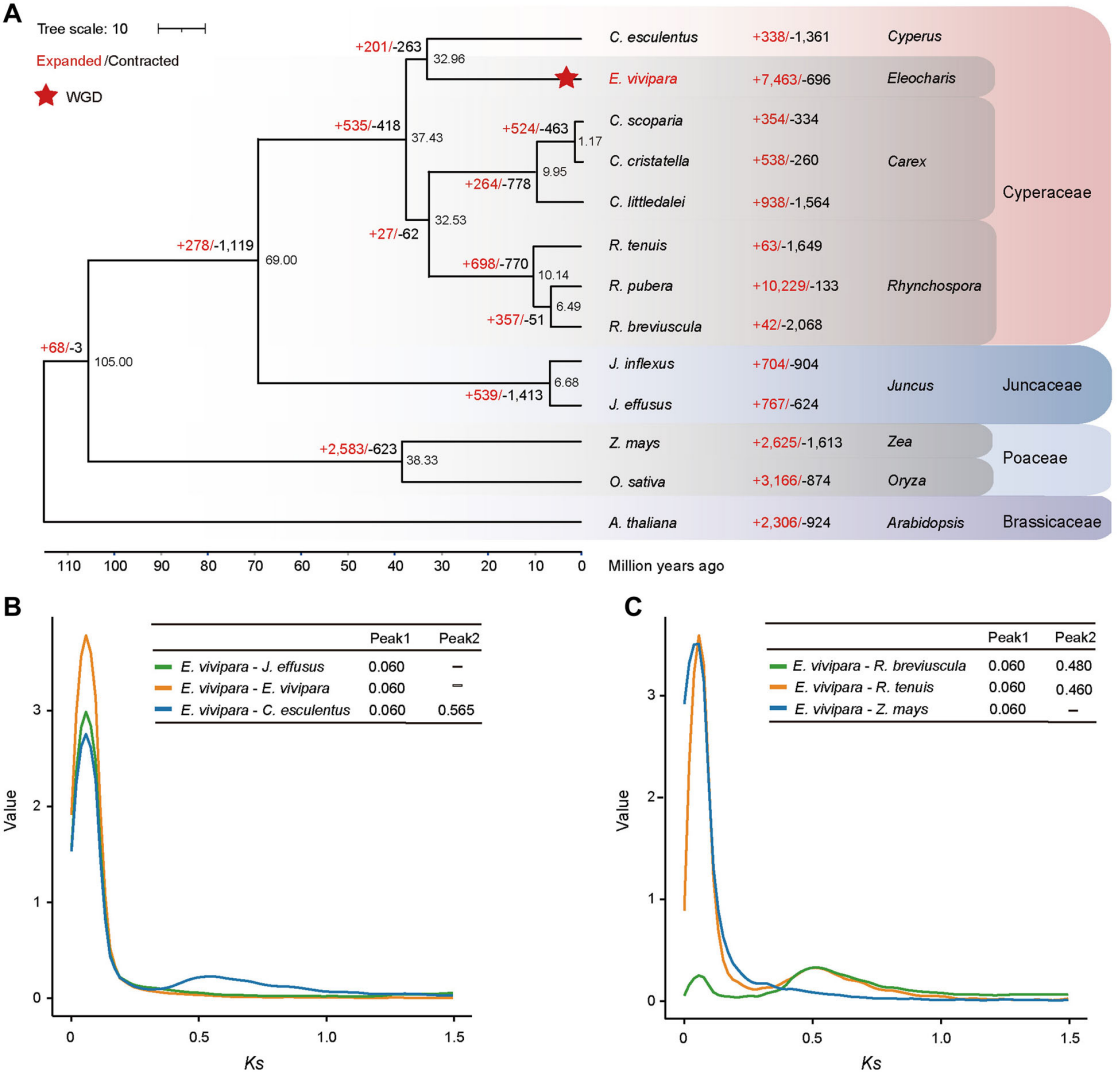
Phylogenetic inference of *Eleocharis vivipara* and other species in Cypearceae **(A)** Whole‐genome phylogenetic tree of *E. vivipara* within Cyperaceae and sister families. Numbers above or below branches refer to the expanded (red) and contracted (black) gene families. Numbers at the node refer to the divergence time for the two branches. **(B**, **C)** Synonymous substitution rates (*K*s) value distribution of homologous genes in *E. vivipara* and other six species. The peak positions of *K*s value distribution are listed in the corresponding tables.

To determine the precise phylogenetic positioning of *E. vivipara*, its 38,769 longest representative proteins were compared with those from 12 other species, including seven sedges, two species each from Juncaceae and Poaceae, and *Arabidopsis thaliana* as outgroup (Ouyang et al., 2007; Lamesch et al., 2012; Can et al., 2020; Hufford et al., 2021; Hofstatter et al., 2022; Planta et al., 2022; Qu et al., 2022; Ning et al., 2023; Zhao et al., 2023). Utilizing Orthofinder2, we found 34,963 of the 38,769 protein‐coding genes in *E. vivipara* were grouped into 14,555 gene families, leaving 3,806 unclassified (Table S8). The phylogenetic tree was constructed with 756 single‐copy genes present in at least nine species (Figure 3A). The Cyperaceae clade has a closer relationship with Juncaceae than the Poaceae family, consistent with recent findings (Larridon et al., 2021). In this dataset, within Cyperaceae, *Eleocharis* and *Cyperus* form a sister clade, while *Carex* and *Rhynchospora* form another that consistent with recent findings (Zhao et al., 2023). According to the referenced divergence times for the genera *Juncus* and *Zea* (confidence interval (CI): 77.70–111.00 Mya), the genera *Juncus* and *Rhynchospora* (CI: 65.60–88.00 Mya) from Timetree (Bremer, 2002; Kumar et al., 2017; Li et al., 2019), we estimated the divergence time between *E. vivipara* and *C. esculentus* was approximately 32.96 Mya (Figure 3A).

Gene family dynamics, including expansion, contraction, and the emergence of unique gene families, are known to shape the traits of species (Feller et al., 2011; Lehti‐Shiu et al., 2017). Analysis with CAFE revealed that after divergence from *C. esculentus*, *E. vivipara* experienced the expansion of 7,463 and contraction of 696 gene families (Figure 3A). The number of expanded gene families was much larger than most other species, but less than that of the hidden octoploid species *R. pubera* (10,229). Strikingly, GO analysis showed these expanded gene families were significantly enriched in terms related to photosynthesis (q‐value = 3.84e‐6), light reactions (q‐value = 3.84e‐6), light harvesting (q‐value = 1.43e‐3), and the photosynthetic electron transport chain (q‐value = 3.71e‐3) (Figure S2A; Table S9A). Moreover, 2,865 of 7,463 gene families were significantly expanded (*P*‐value < 0.01), and they were predominantly involved in protein phosphorylation (q‐value = 6.02e‐6) and regulation of anion transmembrane transport (q‐value = 4.54e‐4), as detailed in Figure S2B and Table S9B. In contrast, the 696 contracted gene families were mainly enriched in response to growth hormone (q‐value = 4.27e‐14) and positive regulation of short‐day photoperiodism and flowering (q‐value = 1.45e‐11) (Figure S2C; Table S9C). The 71 significantly contracted gene families were associated with seed maturation (q‐value = 9.90e‐6), anatomical structure maturation (q‐value = 9.90e‐6), and flower development (q‐value = 4.40e‐4), as shown in Figure S2D and Table S9D. These expanded and contracted gene families likely played roles in *E. vivipara* growth, photosynthesis, and culm development, and likely reflect this species' shift to an amphibious lifestyle (Higgisson et al., 2022).

Within our dataset, *E. vivipara* was found to possess 902 unique gene families when compared with its four closest relatives, *Cyperus esculentus*, *Carex scoparia*, *Carex cristatella*, and *Carex littledalei*. These families are primarily involved in DNA methylation and demethylation (q‐value = 8.17e‐5), regulation of histone H3K27 trimethylation (H3K27me3, q‐value = 5.08e‐4), and positive regulation of transcription elongation (q‐value = 1.20e‐3), as indicated in Figure S2E and Table S9E. Given the recognized role of DNA methylation and H3K27me3 as significant epigenetic mechanisms influencing gene activity (Wang et al., 2009; Moore et al., 2013; Cai et al., 2021; Mattei et al., 2022), it is inferred that these unique gene families may be instrumental in modulating gene expression to facilitate adaptation to the amphibious lifestyle, particularly influencing genes associated with the transition from C_3_ to C_4_ photosynthesis.

### The time of whole‐genome duplication event in *E. vivipara*

To elucidate the time of the whole‐genome duplication (WGD) event in the *E. vivipara* genome, we analyzed the distribution of synonymous substitution rates (*K*s) for its paralogous genes that resulted from the whole and partial genome duplication events, and the orthologous genes between it and other species (Figure 3B, C). The *K*s distribution for orthologous genes between *E. vivipara* and *C. esculentus* peaked at 0.565 (Figure 3B). Using the formula for divergence time, T = *K*s/2r (where r denotes the substitution rate per site per year), and the divergence time of *E. vivipara* and *C. esculentus* approximately 32.96 Mya, we calculated the substitution rate for the Cyperaceae was approximately 8.57 × 1e‐9. This rate is higher than those reported for Poaceae (~6.50 × 1e‐9) and Arecaceae (~2.61 × 1e‐9) (Gaut et al., 1996; SanMiguel et al., 1998), indicating a faster evolutionary rate in the Cyperaceae. The peak *K*s values for gene pairs in *E. vivipara* versus *Rhynchospora breviuscula* or *R. tenuis* were 0.480 and 0.460, respectively (Figure 3C), much larger than that of *E. vivipara* with itself at 0.060, suggesting that the divergence time for these species occurred earlier than the WGD event in *E. vivipara*, consistent with genome synteny results. Similarly, the *K*s values of ortholog genes between *E. vivipara* and other species all displayed peaks at 0.060. Based on the formula, we estimated the WGD event in *E. vivipara* occurred approximately 3.5 Mya (Figure 3A), indicating a relatively recent event, consistent with its large number (7,463) of expanded gene families.

### Chromosome fusion and fission in Cyperaceae

**Figure 4:**
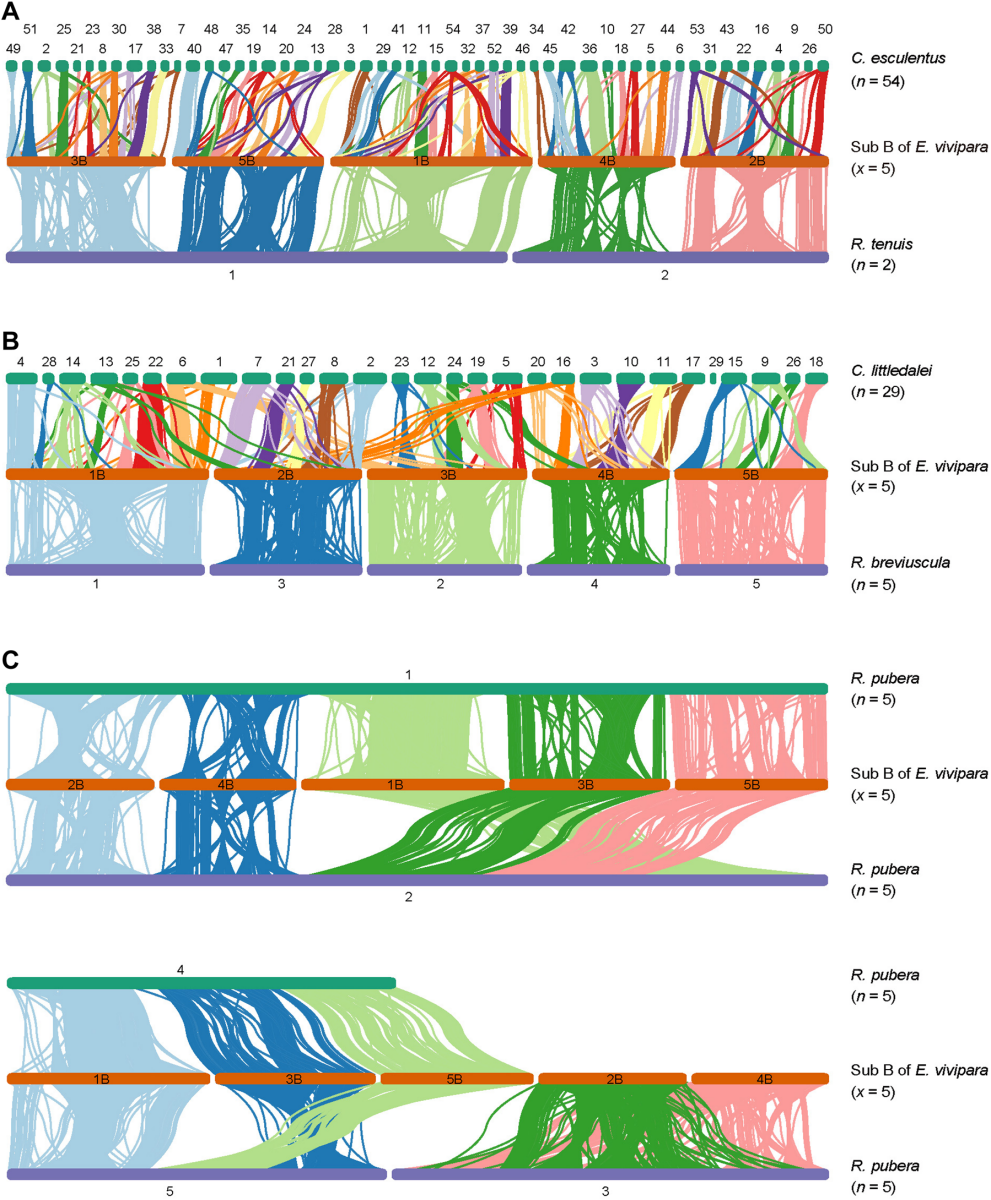
The genome synteny map for *Eleocharis vivipara* and its close relatives **(A)** The one‐to‐one synteny relationship between the subgenome B of *E. vivipara* (*x* = 5) and the genome of *Cyperus esculentus* (*n* = 54) and *Rhynchospora tenuis* (*n* = 2), with the former displaying frequent chromosome fission events and the latter displaying end‐to‐end fusion events, respectively. **(B)** The one‐to‐one relationship between subgenome B of *E. vivipara* (*x* = 5) and the genome of *Carex littledalei* (*n* = 29) and *Rhynchospora breviuscula* (*n* = 5), with *C. littledalei* also displaying frequent chromosome fission events. **(C)**. The one‐to‐four relationship between subgenome B of *E. vivipara* (*x* = 5) and the hidden octoploid level genome of *Rhynchospora pubera* (*n* = 5), with the latter also displaying frequent chromosome end‐to‐end fusion events.

Genome synteny analysis was used to investigate karyotype evolution in the Cyperaceae family which exhibits a wide chromosomal range with *n* = 2 to 113 (Roalson, 2008). Here we compared the genome of *E. vivipara* with its close relatives, including *C. esculentus* (*n* = 54) in *Cyperus*, *C. littledalei* (*n* = 29) in *Carex*, and *R. pubera* (*n* = 5), *R. breviuscula* (*n* = 5), and *R. tenuis* (*n* = 2) in *Rhynchospora* (Table 1). *C. esculentus* has the maximum chromosome number in the published genomes of Cypearceae, and *R. tenuis* has the minimum number known in plants (Hofstatter et al., 2022; Zhao et al., 2023). Initially, we examined the synteny of paralogous genes within the two subgenomes of *E. vivipara*, revealing a clear one‐to‐one coordinate relationship, albeit with significant chromosome inversions between Chr1A and Chr1B (Figure 2H). Further comparative analyses demonstrated that the genome of *C. esculentus* (*n* = 54) and *C. littledalei* (*n* = 29) have substantial one‐to‐one synteny with *E. vivipara* subgenome B (Figure 4A, B). Numerous chromosome fission events, resulted in the chromosome number increasing ~11 and ~6 times, respectively. In contrast, the genome of *R. tenuis* (*n* = 2) and *R. pubera* (*n* = 5) displayed one‐to‐one and four‐to‐one relationship with the subgenome B (Figure 4A, C), which not only supported the result that *R. pubera* has a hidden octoploid level, but also displayed both had experienced end‐to‐end chromosome fusion events. In addition, the intact one‐to‐one synteny relationship between the genome of *R. breviuscula* (*n* = 5) and the subgenome B of *E. vivipara* supported the previous finding that the ancestral chromosome number of *Rhynchospora* genus is *n* = 5 (Figure 4B) (Burchardt et al., 2020). Collectively, these findings provided a systematic model of karyotype evolution within the Cyperaceae family and explained the speciation events.

### *E. vivipara* has holocentromeres that can maintain the stability of chromosomes after fission or fusion

Holocentromeres, unlike monocentromeres, have been reported to stabilize chromosome structure following fusion or fission events, thereby facilitating karyotype rearrangements and potentially speciation (Monti et al., 2012; Manicardi et al., 2015; Lukhtanov et al., 2018). To characterize the centromere structure of *E. vivipara* chromosomes in the context of the numerous fusion and fission events compared to its close relatives, we examined the spatial distribution of its gene expression, Hi–C interaction intensity, and the density of repeats and genes on single chromosome. These features were then contrasted with *Juncus effusus* which has monocentromeres (Hofstatter et al., 2022; Planta et al., 2022).

**Figure 5:**
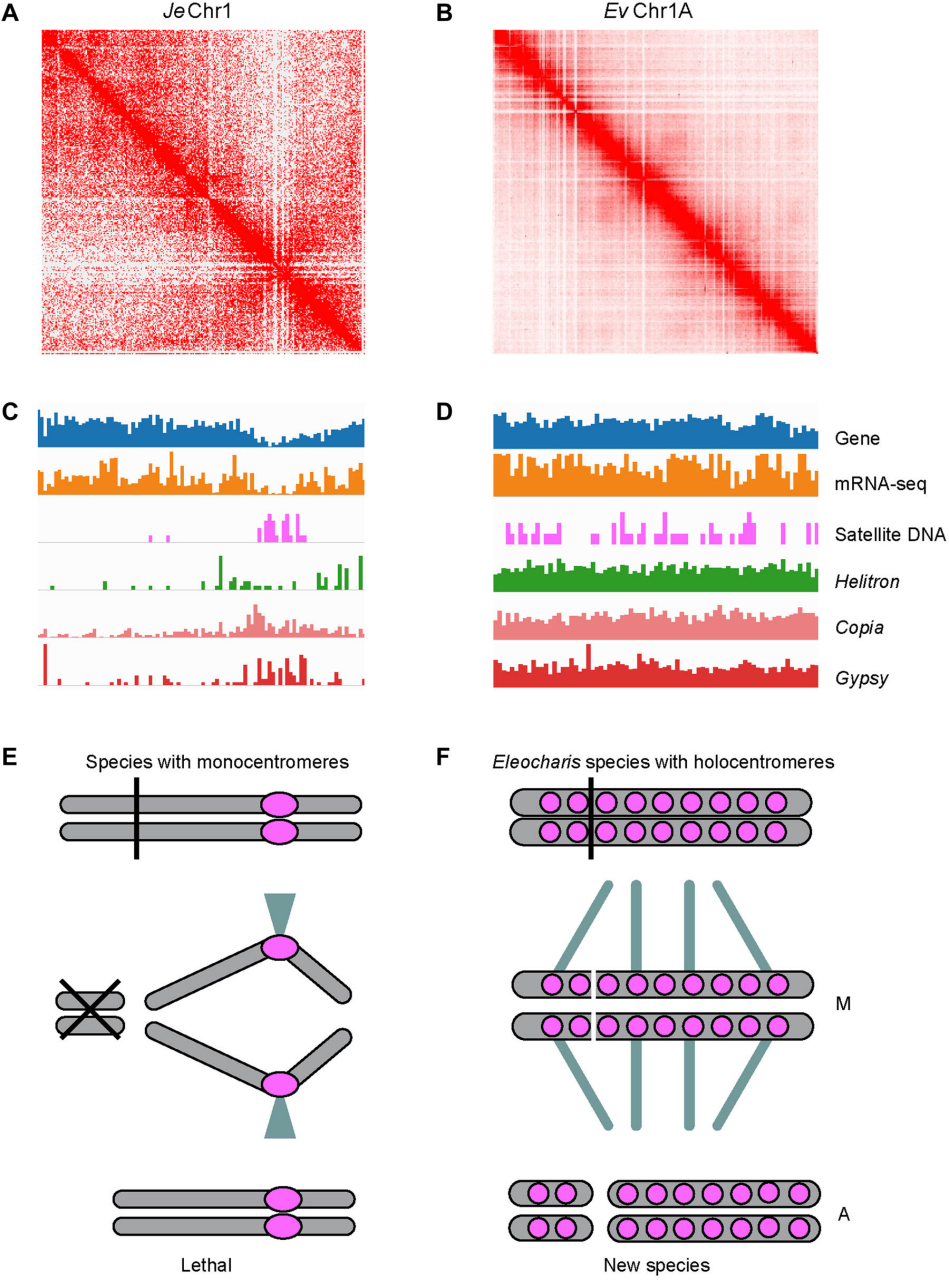
Comparison of spatial genome organization in *Eleocharis vivipara* and *Juncus effusus* **(A**, **B)** Hi–C interaction map for Chr1 of the *J. effusus* and Chr1A of the *E. vivipara* genomes, with their length are 14.09 Mb and 116.72 Mb, respectively. **(C**, **D)** The distribution of genes, gene expression and different repeats in the same chromosome. The window size for **(C)** and **(D)** are 150 kb and 1 Mb, respectively. **(E**, **F)** The effect of monocentromeres (left) and holocentromeres (right) on fission chromosomes, respectively. The former may lead to chromosome separation problems during meiosis and cause lethality **(E)** while the latter can maintain the stability of fission chromosomes and prompt speciation **(F).** The purple circle indicates the centromere position. M, metaphase. A, anaphase.

In *J. effusus*, the Hi–C interaction intensity map along a single chromosome exhibits an X‐shape, indicative of extensive contact in a telomere‐to‐centromere axis, with euchromatin and heterochromatin compartments clearly distinguishable (Figures 5A, S3), typical of the monocentromeric chromosomes found in most plants. Its gene density and expression both decrease significantly in the centromeric region compared to the rest of the chromosome (Figure 5C). Additionally, its satellite DNA is concentrated in the centromeres, with LTR/*Copia*, LTR/*Gypsy* retrotransposons, and DNA transposons primarily accumulating in centromere and pericentromere regions.

In contrast, the Hi–C pattern of *E. vivipara* genome exhibits no large‐scale compartmentalization, and its interaction intensity pattern shows a lack of prominent contacts between telomeres and centromeres (Figures 1C, 5B). The *E. vivipara* genome demonstrates a uniform and continuous distribution pattern of gene density and expressions, satellite DNA, LTR/*Copia*, LTR/*Gypsy* retrotransposons, and *Helitron* transposons across all the whole chromosome (Figures 2C–G, 5D). These observations indicate that the chromosomes of *E. vivipara* exhibit typical holocentromere characteristics in their spatial genome organization, a pattern similar to that observed in recently published genomes of species in *Rhynchospora* (Hofstatter et al., 2022), and markedly distinct from *J. effusus* and most other plants with monocentromeres.

For the species like *J. effusus* which have monocentromeric chromosomes, their chromosomes are prone to have abnormal or defective centromere activity following fusion or fission, leading to problems with chromosome segregation in metaphase and anaphase during meiosis, which can result in sterility or lethality (Figure 5E). In contrast, given that the *Eleocharis* genus encompasses approximately 252 species with varying chromosome numbers (e.g., 2*n* = 10, 16, 18, 20, 36, 38, 40) (Zedek et al., 2010), we hypothesize that holocentromeres in *Eleocharis* play a crucial role in permitting normal meiosis following chromosome rearrangement, and potentially facilitating speciation (Figure 5F).

### Dynamic Kranz anatomy and gene expression patterns associated with C_3_ and C_4_ photosynthetic plasticity

**Figure 6:**
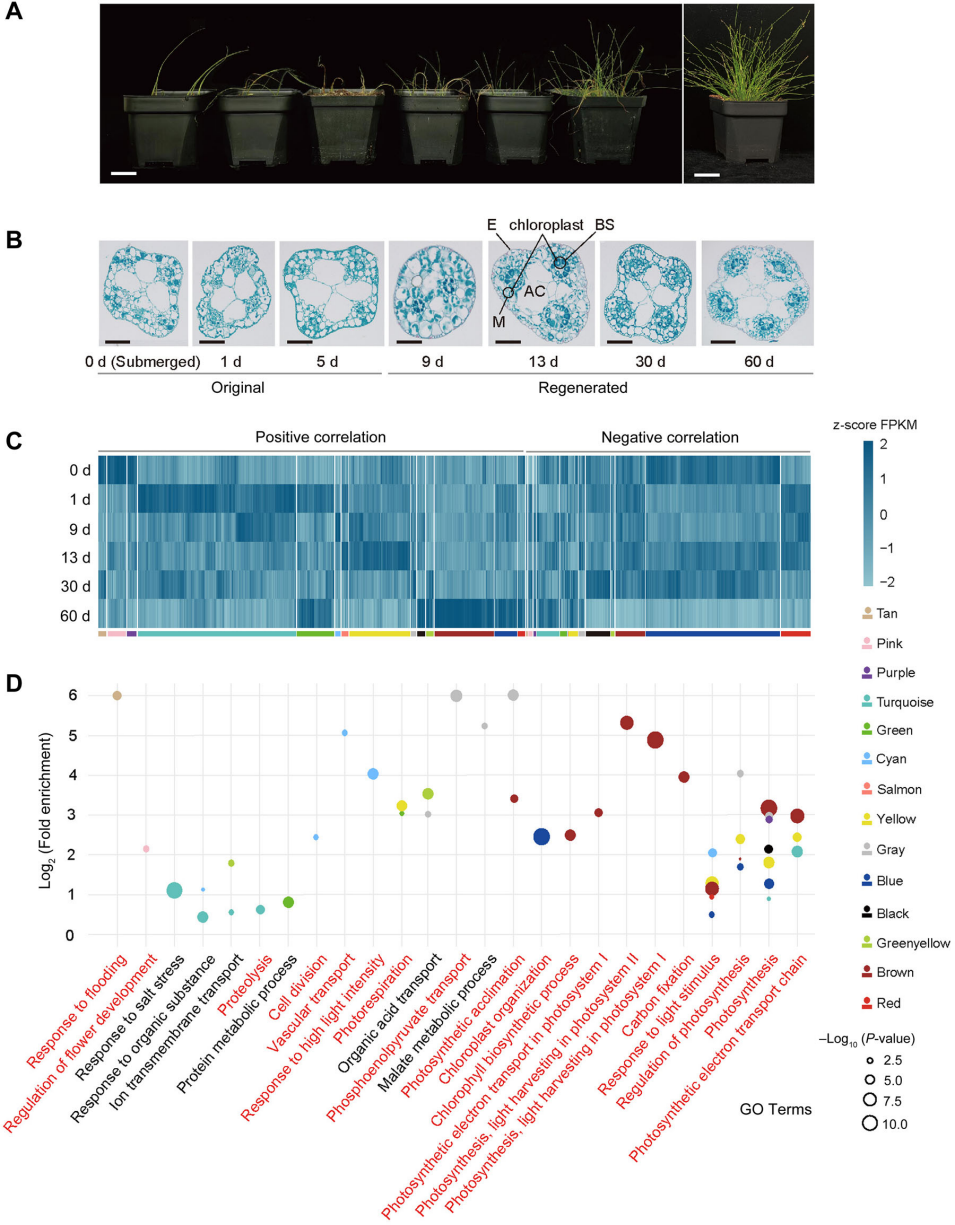
Anatomical and dynamic gene expression patterns in submerged versus terrestrial culms of *Eleocharis vivipara* **(A**, **B)**. Whole‐plant phenotype and culm anatomy changes of *E. vivipara* after transplantation 1, 5, 9, 13, 30, and 60 d from submerged (0 d) to terrestrial conditions. Scale bars for **(A)** represent 3 cm. For **(B)**, from 0 to 30 d, 0.25 mm; and for 60 d, 0.30 mm. BS, bundle sheath (BS) cells; M, mesophyll (M) cells; E, epidermis; AC, air cavity. **(C)** Gene expression differences between the culms of the six different stages. Here 14 of the 15 modules in weighted gene co‐expression network analysis (WGCNA) results showed significant correlation with at least one of the six stages based on *P*‐value < 0.01. **(D)** Gene Ontology functional analysis of the significantly related modules. The biological processes related to photosynthesis and characteristics of each stage during the transition from C_3_ to C_4_ are highlighted in red font.

To uncover the molecular mechanisms underlying *E. vivipara*'s adaptation to amphibious habit, we conducted a transplanting experiment and comparative transcriptome analysis. Upon transferring *E. vivipara* from submerged to terrestrial conditions, initial severe withering was observed from d 1 to d 5 post‐transplant, followed by regeneration on d 9 to d 13, and then robust growth by d 30 and 60, demonstrating remarkable regenerative capability. Phenotypically, the regenerated terrestrial culms were erect and firm, in contrast with the slender and soft submerged culms (Figure 6A). Cross‐sectional analysis revealed that the regenerated culms gradually developed typical Kranz anatomy, a hallmark of C_4_ photosynthesis. Additionally, chloroplast density in the BS cells dramatically increased (Figure 6B). These findings demonstrate the substantial differences in photosynthetic phenotype and associated anatomy and ultrastructure between *E. vivipara* culms in the two environments, with the terrestrial environment inducing C_4_ Kranz anatomy.

To investigate the molecular basis of *E. vivipara'*s transition from submerged to terrestrial environments, and from C_3_ to C_4_ photosynthesis, we collected the living culms of submerged (0 d) and terrestrial states (1, 9, 13, 30, and 60 d after transplantation), sequenced transcriptomes for all samples, and conducted comparative analysis. A total of 31,939 expressed genes (fragments per kilobase of exon per million reads mapped (FPKM) >1.0) were identified in these culms, with each biological replicate expressing between 21,549 and 28,534 genes (Table S10). According to the three‐dimensional principal component analysis (3D‐PCA) result, we found that PC1, PC2, and PC3 explained 31.4%, 17.8%, and 15.2% of observed variation, respectively. Different overall gene expression patterns were also observed among different transitional stages (Figure S4A). To identify genes which associate with C_3_ and C_4_ photosynthetic plasticity, we conducted the weighted gene co‐expression network analysis (WGCNA). We filtered 6,000 expressed genes with the best median absolute deviation (MAD) values, and found they could be well clustered into 15 modules (Figure S4B), 14 of which showed significant correlation with at least one of the six transitional stages (*P*‐value < 0.01) (Figures S4B, 6C).

Interestingly, after transplantation of *E. vivipara* culms from a submerged to terrestrial state, we found that photosynthesis‐related processes were enriched through all stages, especially for the response to light stimulus, regulation of photosynthesis, photosynthesis, and photosynthetic electron transport chain, highlighting the importance of photosynthesis to the transition from submerged to terrestrial conditions (Figure 6D). Here, the representative gene in regulation of photosynthesis is proline‐tRNA ligase‐like protein (also known as proline‐tRNA synthetase). This gene is related to proline biosynthesis, a process that can consume photosynthesis generated NAD(P)H to regenerate NAD(P)^+^ to sustain photosynthetic electron transport (Figure S5A) (Alvarez et al., 2022). Increased proline accumulation was also reported to promote photosynthesis under stress (Surender Reddy et al., 2015). Strikingly, the expanded gene families in *E. vivipara* include 92.16%, 47.46%, and 95.65% of genes here involved in photosynthesis, light stimulus, and light harvesting, respectively (Figure S6A–C), providing strong evidence for its contribution to the transition of photosynthesis types.

We also identified important GO terms that significantly enriched (q‐value < 0.05) by the expressed genes which significant correlated with each transitional stage. In the 0 d (submerged) state, the response to flooding (q‐value = 2.00e‐2) and regulation of flower development (q‐value = 2.34e‐2) were enriched, coordinated with the phenotype of more and new culms in submerged plants, a consequence of proliferation from sterile spikelets at the culm apex (Figures 6D, S7). In the 1 d state, protein metabolic process (q‐value = 3.07e‐5) and proteolysis (q‐value = 3.30e‐3) were significantly enriched, corresponded to the withering plant phenotype. In the early stages after regeneration (9 and 13 d culms), cell division (q‐value = 2.31e‐2), vascular transport (q‐value = 2.07e‐2), and photorespiration (q‐value = 1.67e‐3) were significantly enriched, reflecting its newly developed Kranz anatomy. Here in the cell division network, the ethylene responsive factor (ERF) transcription factors are representative genes that can regulate the divisions of vascular cells (Figure S5B) (Etchells et al., 2012). Photorespiration is exacerbated by high temperature, high light intensity, and drought, and is thought to be a major driver of C_4_ evolution (Wingler et al., 2000; Mallmann et al., 2014; Heyduk et al., 2019). Here the glycolate oxidase (GLO/GOX) gene is a representative one which encodes a crucial enzyme in the photorespiration pathway and catalyzes the conversion of glycolate into glyoxylate (Figure S5C) (Foyer et al., 2009). In the latter stage (30 d and 60 d culms), the terms of photosynthetic acclimation (q‐value = 7.43e‐4), phosphoenolpyruvate (PEP) transport (q‐value = 8.86e‐5), chloroplast organization (q‐value = 1.60e‐27), chlorophyll biosynthesis process (q‐value = 7.21e‐4), light harvesting in photosystem I (q‐value = 5.40e‐28) and II (q‐value = 1.66e‐6), and carbon fixation pathway (q‐value = 6.73e‐4) were significantly enriched (Figure 6D), corresponding to a fully functional C_4_ photosynthetic pathway with increased carbon fixation capability. Here in the chlorophyll biosynthetic network, chlorophyllide a oxygenase (CAO) is a representative gene which is responsible for converting chlorophyll a to b (Figure S5D) (Dey et al., 2023). Chlorophyll b is a main component of the light‐harvesting complex and helps in absorbing a diverse range of light spectra for photosynthesis (Chen, 2014). In the carbon fixation network, sedoheptulose‐1,7‐bisphosphatase (SBPase) is the representative gene which encodes an important enzyme involved in photosynthetic carbon fixation in the Calvin cycle, and its increased activity can result to increased photosynthetic capacity (Figure S5E) (Ding et al., 2016). Together, these dynamic patterns reflect the molecular factors controlling the regeneration of new culms, development of Kranz structure, and elevated carbon fixation ability after transplantation of *E. vivipara* plants.

### Other drivers related to photosynthetic plasticity

To fully capture the drivers of the photosynthesis plasticity, we also compared the transcriptome of the terrestrial culms (30 d) with that of submerged (0 d), and identified their DEGs. Applying strict criteria of fold‐change >2, q‐value < 0.05, and FPKM > 1, we identified 2,602 upregulated DEGs (up‐DEGs) and 1,095 downregulated DEGs (down‐DEGs) in the terrestrial culms. To verify the expression of the genes, we also compared the 13 d growing culms with the submerged, then selected nine DEGs and two non‐DEGs as control, and validated by reverse transcription quantitative polymerase chain reaction (RT‐qPCR) (Table S11). The RT‐qPCR result showed good consistency with the messenger RNA sequencing (mRNA‐seq) data (*R* ^2^ = 0.91, *P*‐value = 2.86e‐9) (Figure S8A), and well validated the identified DEGs (Figure S8B).

Gene Ontology enrichment analysis of the 2,602 up‐DEGs revealed significant enrichment in terms related to ion transmembrane transport (q‐value = 6.85e‐19), response to water deprivation (q‐value = 5.17e‐11), hormone metabolic processes (q‐value = 2.24e‐7), and response to abscisic acid (ABA, q‐value = 5.24e‐7) (Figure S9A; Table S12A). Water stress, in addition to CO_2_ concentration, is recognized as a key evolutionary driver for C_4_ plants which typically exhibit higher water use efficiency than C_3_ plants (Edwards et al., 2010; Westhoff and Gowik, 2010; Way et al., 2014; Pardo and VanBuren, 2021; Ozeki et al., 2022). ABA may act as a signaling molecule to integrate many responses to water, as it can induce Kranz anatomy and C_4_‐like biochemical characteristics in submerged *E. vivipara* plants (Ueno, 1998). Consequently, the terrestrial environment may trigger Kranz anatomy development by activating the 98 up‐DEGs associated with water deprivation and the 107 up‐DEGs responsive to ABA (Figure S10A, B). Notably, among the 98 up‐DEGs, six genes encode 9‐cis‐epoxycarotenoid dioxygenase and two genes encode ABA 8′‐hydroxylase‐like proteins, both involved in ABA biosynthesis and metabolism (Saito et al., 2004; Huang et al., 2019), respectively, indicating these genes may have dual roles in water deprivation and ABA metabolism in *E. vivipara*. The 98 up‐DEGs involved in response to water deprivation also included genes that are well known to regulate drought tolerance, including six genes encoding NAC domain‐containing protein (Puranik et al., 2012; Kurowska and Daszkowska‐Golec, 2023), two genes encoding E2 ubiquitin‐protein ligase PUB22‐like protein (Cho et al., 2008), and one gene encoding a guard cell S‐type anion channel SLAC1‐like (slow anion channel‐associated 1) protein (Vahisalu et al., 2008). Interestingly, SLAC1 was found preferentially expressed in guard cells and is essential for stomatal closure in response to CO_2_, ABA, and changes in humidity (Vahisalu et al., 2008). Reduced stomatal aperture and density have been identified as important modifications during C_4_ evolution (Zhao et al., 2022).

Conversely, the 1,095 down‐DEGs were primarily enriched in the regulation of flower development (q‐value = 1.71e‐6), response to stimulus (q‐value = 2.04e‐7), and transcription factor activity (q‐value = 3.25e‐6) (Figure S9B; Table S12B). Within the set of transcription factors, 21.5% (16 out of 76) of the genes encode ERFs (Figure S10C), which may respond to ethylene, cytokinin, and auxin, influencing stomatal density, photosynthesis, and growth (Upadhyay et al., 2013).

Together, these results suggested that the development of Kranz anatomy and onset of C_4_ photosynthesis in terrestrially grown culms of *E. vivipara* require coregulation of other various factors, particularly water deprivation, response to ABA, and transcription factor‐related regulation.

### The evolution of C_4_ photosynthesis predates the polyploidization event in *E. vivipara*

**Figure 7:**
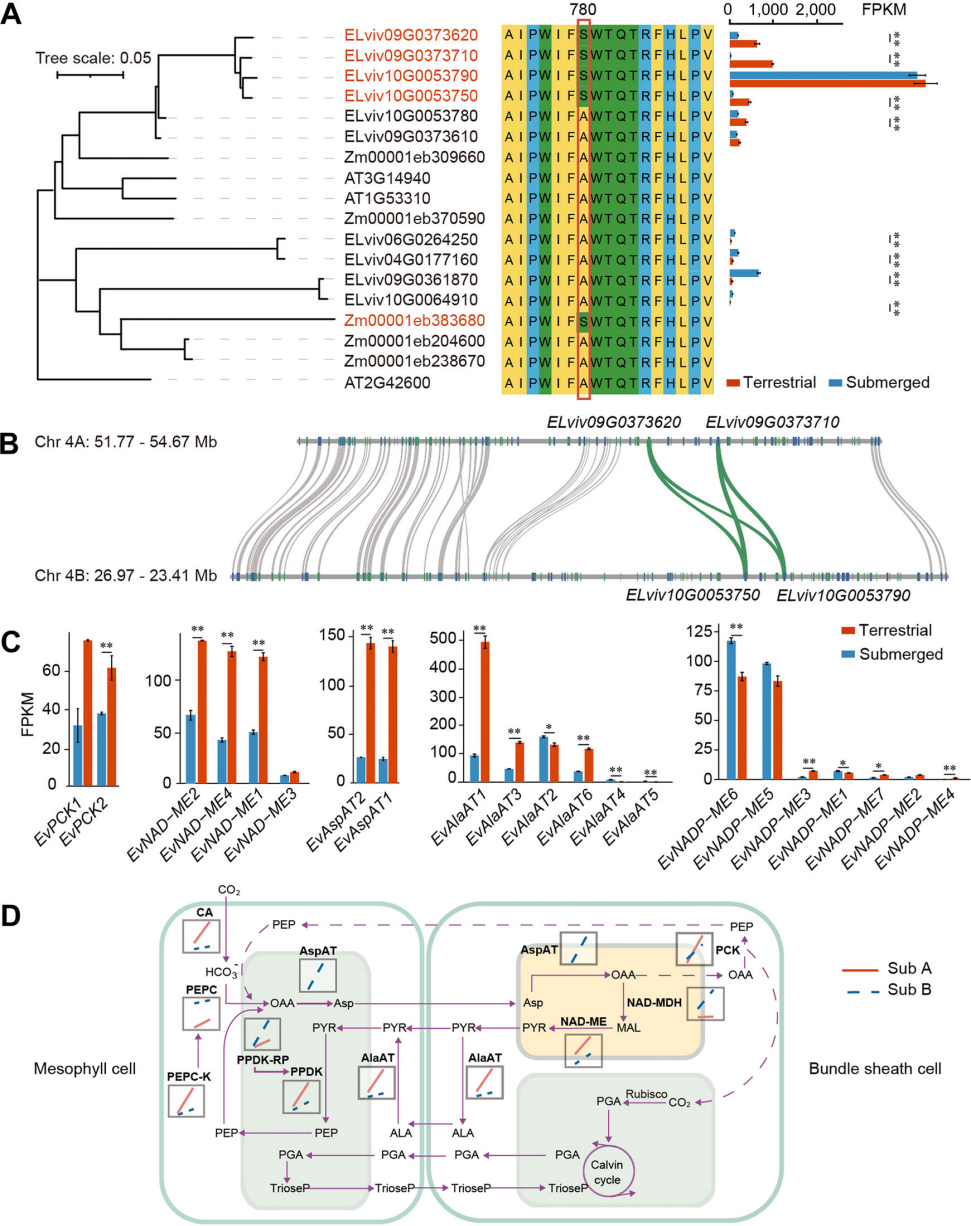
Evolution and expression differences for the genes involved in C_4_ core pathway between submerged and terrestrial culms of *Eleocharis vivipara* **(A)** The phylogeny, protein alignment and expression level of phosphoenolpyruvate carboxylase (PEPC) genes in *E. vivipara*. The PEPC genes responsible for C_4_ core pathway are highlighted with red color, and the outgroup sequence is the bacterial‐type PEPC in *Arabidopsis thaliana*. The bootstrap values for the phylogeny tree ranged from 31 to 100. The position 780 in the C_4_‐adapted PEPC protein in maize is labeled with red frame. For the bar plot, * indicate q‐value < 0.05; ** indicate q‐value < 0.01. **(B)** Gene synteny for the regions in Chr4A: 51.77–54.67 Mb and Chr4B: 26.97–23.41 Mb. The PEPC genes responsible for the C_4_ pathway are highlighted with green lines, with the genes *ELviv09G0373620* and *ELviv09G0373710* are in subgenome A, and *ELviv10G0053790* and *ELviv10G0053750* are in subgenome B, respectively. **(C)** The expression difference of phosphoenolpyruvate carboxykinase (PCK), nicotinamide adenine dinucleotide ‐ malic enzyme (NAD‐ME), aspartate transaminase (AspAT), alanine transaminase (AlaAT), and NAD phosphate (NADP)‐ME genes in submerged and terrestrial culms. *, q‐value < 0.05; **, q‐value < 0.01. **(D)** Schematic of the C_4_ pathway in *E. vivipara*. Genes responsible for C_4_ core pathway were identified in subgenome A (red) and B (blue), and their expression (fragments per kilobase of exon per million reads mapped) levels before and after transplantation are plotted in the box. For **(A**, **C** and **D)**, the submerged and terrestrial represent 0 d and 30 d culms after transplantation, respectively.

To identify which phosphoenolpyruvate carboxylase (PEPC) genes in *E. vivipara* are responsible for assimilating atmospheric CO_2_ during C_4_ photosynthesis, we conducted phylogenetic analysis and aligned their protein sequences. Orthofinder identified 10 genes encoding PEPC in *E. vivipara* (Figure 7A), while further alignment of protein sequences identified four of them as putative C_4_ isoforms, according to presence of a signature substitution of alanine (A) for serine (S) at position 780 in the C‐terminal end of C_4_ PEPC protein in maize. This substitution is considered as a major determinant of affinity for PEP (Bläsing et al., 2000; Christin et al., 2007). The four putative C_4_ isoforms are evenly distributed in subgenome A and B, respectively (Figure 7B), suggesting that C_4_ photosynthesis predates the polyploidization event. The result is different from that found in most species, such as maize, which has only one C_4_ isoform. Moreover, among the 13 species in the phylogenetic tree, we also found that the C_4_ species *C. esculentus* has one C_4_‐adapted PEPC copy with similar characteristics (Figure S11) (Larridon et al., 2013; Zhao et al., 2023). However, the *Cyperus* genus also contains many C_3_ species, and the phylogenetic position of the C_4_ PEPC gene in *C. esculentus* is distinct from those in *E. vivipara*, reflecting the independent origins of C_4_ photosynthesis in *Eleocharis* and *Cyperus* (Sage et al., 2011; Larridon et al., 2013). Therefore, we estimated the evolution time for C_4_ photosynthesis in *E. vivipara* occurred between 32.96 and 3.5 Mya, based on the divergence time between *E. vivipara* and *C. esculentus* and the WGD event in *E. vivipara*. Given the low taxon sampling and the large phylogenetic distance between *Eleocharis* and *Cyperus*, it is likely much closer to the smaller estimate of 3.5 Mya.

To ascertain which amino acids have been subject to positive selection in C_4_ PEPC genes across Cyperaceae, Juncaceae, Poaceae, and Brassicaceae families (Figure S11), we detected their posterior probability (PP) value in these PEPC genes. We found the well‐studied A to S modification at position 780, together with 409, 665, and 731 all showed PP values > 0.99, while positions 14, 350, 460, 501, 572, 584, and 770 had PP values between 0.95 and 0.99, indicated they were all under significant positive selection. These amino acids provide the molecular signals of C_4_‐adapted PEPC for the convergent evolution of C_4_ in different species of Cyperaceae and Poaceae families (Sage et al., 2011).

Interestingly, one of the four putative C_4_‐adapted PEPC genes in *E. vivipara* exhibited much higher expression (FPKM > 2,200) than the other three (FPKM < 600), but no significant change in terrestrial culms compared with submerged conditions (Figure 7A). Although the other three were expressed at lower levels (FPKM > 200), they showed significant up‐regulation in terrestrial culms. These results indicate the four C_4_‐adapted PEPC genes in *E. vivipara* respond differently during acclimation to terrestrial conditions, a marked difference from most C_4_ species which have just one C_4_ isoform, or tandem duplications of PEPC with similar expression patterns to increase the gene dosage (Bianconi et al., 2018).

### C_4_ photosynthesis in *E. vivipara* utilizes mixed NAD‐ME and PCK subtypes

To further elucidate which subtype of C_4_ photosynthesis was employed by the terrestrial culm of *E. vivipara*, we identified and compared the expression of genes encoding NAD‐ME, NADP‐ME, PCK, together with the aspartate transaminase (AspAT) and alanine transaminase (AlaAT), with the latter two genes known to be primarily involved in the NAD‐ME pathway (Rao et al., 2016). Compared with the submerged type, in terrestrial culms, two PCK (FPKM > 60) and three NAD‐ME genes (FPKM > 120) exhibited up‐regulation (Figure 7C), along with two AspAT (FPKM > 140) and three AlaAT (FPKM > 110) genes. Only two of the seven genes encoding NADP‐ME were highly expressed (FPKM > 80), but exhibited decreased expression in terrestrial culms, while the others showed relatively low expression (FPKM < 10). These results provided expression evidence that the terrestrial culm of *E. vivipara* utilizes a mixture of NAD‐ME and PCK C_4_ pathways, rather than NADP‐ME or just NAD‐ME (Ueno et al., 1988), and it showed overall elevated expression of core genes associated with the C_4_ pathway (Figures 7A, C, S12).

To explore the two subgenomes' contributions to the core C_4_ pathway, we then compared their gene expression levels (Figures 7D, S12). In terrestrial culms, compared with the subgenome B, we found that the genes encoding PCK, NAD‐ME, AlaAT, β‐carbonic anhydrase (CA), PEPC kinase (PEPC‐k), and pyruvate orthophosphate dikinase (PPDK) showed higher expression levels in subgenome A (Figure 7D; Table S13), indicating subgenome A contributes more than B in determining the subtypes of C_4_ in *E. vivipara*. However, for the genes encoding the PEPC, PPDK regulatory protein (PPDK‐RP), AspAT, malate dehydrogenase (NAD‐MDH), they showed much lower expression in subgenome A, indicating the subgenome B provides a complementary role in the C_4_ pathways. This mixed contribution of genes located in both subgenomes to C_4_ photosynthesis demonstrates that both subgenomes contribute flexibly to C_4_ function, and this aligns with our hypothesis that the C_4_ phenotype evolved prior to the WGD event in *E. vivipara*.

## DISCUSSION

Although the capacity for switching between C_3_ and C_4_ photosynthesis in *E. vivipara* is of high value as a model for the study of the genetic control of C_4_ photosynthesis, this promising area of research has been hampered by lack of sequenced genomes. Here we present the high‐quality reference genome assembly of *E. vivipara*, representing the first chromosome‐level genome of the *Eleocharis* genus, and particularly with regard to questions of genetic control of C_4_ photosynthesis. Based on the five pairs of strong interaction in the Hi–C map (Figure 1C), the chromosome cluster result of subgenomes A and B (Figure 1D), and the one‐to‐one genome synteny relationship between the two subgenomes (Figure 2H), we discovered *E. vivipara* is tetraploid (Table 1). The assembled genome is 965.22 Mb, and contains 38,769 predicted protein‐coding genes, which is 1.42 fold higher than was recently reported (27,249 genes) based on the *de novo* assembly of short‐read data (Harada et al., 2018). Phylogenetic analysis using nuclear‐encoded gene families revealed a closer relationship with the *Cyperus* genus than others in our dataset, indicating a divergence time of approximately 32.96 Mya (Figure 3A). The identified 7,463 expanded, 696 contracted, and 890 unique gene families in *E. vivipara* may enhance its adaptability to an amphibious lifestyle (Figure S2; Table S9). Additionally, genome synteny comparisons with other species highlighted frequent chromosomal end‐to‐end fusion and fission events, resulting in a large chromosome number variation between two and 54 in Cyperaceae (Figure 4). These fusions alongside other chromosomal rearrangements, such as inversions and translocations, likely played a role in the evolution of *E. vivipara* and speciation within the Cyperaceae family which contains ~5,400 species (Roalson, 2008).

Previous studies have suggested that the stability of fusion or fission chromosomes can be maintained by holocentromeres, which may also influence 3D‐genome architecture by altering genomic compartmentalization (Hofstatter et al., 2022). In our findings, *E. vivipara*'s genome structure exhibited holocentromeric characteristics, evidenced by a uniform distribution of Hi–C interaction intensity, as well as *Copia*, *Gypsy*, *Helitron*, satellite DNA, gene density, and gene expression (Figure 5B, D). This pattern mirrors that of the recently sequenced *R. pubera* genome with holocentromeres, and markedly differs from the outgroup *J. effusus* in Juncaceae (Figure 5A, C). The fundamental unit of holocentromeres in *R. pubera* chromosomes has been identified as the 172 bp Tyba repeat (Marques et al., 2015; Hofstatter et al., 2022), yet its specifics in *E. vivipara* remain to be elucidated. Future studies can integrate additional techniques such as CENH3 ChIP‐seq (chromatin immunoprecipitation followed by sequencing), Cut and Tag experiments, and immuno‐FISH (immunolabelling and fluorescence *in situ* hybridization) (Park, 2009; Marques et al., 2015; Deng et al., 2022), to further delineate the exact position and composition of holocentromeres in *E. vivipara*. Nonetheless, the high‐quality, chromosome‐level genome presented here, coupled with comparable assemblies of its close relatives, can serve as foundational models for examining genome architecture evolution, spatial organization, and the mechanisms of speciation for the *Eleocharis* genus and the wider Cyperaceae family (Figure 5F), characterized by their diverse chromosome numbers (Roalson, 2008).

*E. vivipara* exhibits a remarkable amphibious lifestyle characterized by high photosynthetic plasticity, switching between the C_3_ and C_4_ photosynthetic phenotypes in aquatic and terrestrial environments, respectively. This contrasts with the overwhelming majority of C_4_ species, which are entirely obligate in their use of the C_4_ pathway. Apart from *E. vivipara*, this co‐occurrence of C_3_ and C_4_ photosynthesis within a single individual in different organs or at different stages of the life cycle is only known in a handful of amphibious grass species in the Orcuttieae subtribe within the Chloridoideae (Keeley, 1998), and in C_4_ eudicots in the Chenopodiaceae tribe Salsoleae (Pyankov et al., 2000; Lauterbach et al., 2017). This study presents the first high‐quality reference genome for any plant species capable of such mixed photosynthetic pathway utilization, and thus provides an invaluable complement to lineages rich in C_3_–C_4_ intermediate phenotypes, such as *Alloteropsis*, *Flaveria*, *Blepharis*, and *Cleome*, for the study of the evolution and genetic control of C_4_ photosynthesis (Koteyeva et al., 2011; Fisher et al., 2015; Lundgren et al., 2016; Dunning et al., 2019; Stata et al., 2019; Adachi et al., 2023). Our transplanting and sectioning experiments revealed stable differences in phenotype and anatomical structure between the two types of *E. vivipara* culms (Figure 6A, B). Moreover, WGCNA and DEGs analysis results not only displayed the importance of genes involved in light stimulus, photorespiration, water deprivation, and ABA during the C_4_ photosynthesis transition (Figures 6C, D, S9), but also revealed increased expression of specific genes related to the core C_4_ pathway, and significant enrichment of genes related to cell division, chlorophyll biosynthetic, regulation of photosynthesis, and carbon fixation. These results correspond to the development of Kranz anatomy and increased photosynthetic efficiency. A detailed study of key hub genes identified here in the regulatory network will undoubtedly provide important insight into the genetic control of C_4_ photosynthesis and the broader transition to a terrestrial environment in *E. vivipara*.

It is hypothesized that the expression of the key genes may be governed by epigenetic modifications, such as DNA methylation and H3K27 trimethylation, influenced by environmental conditions and stresses (Cai et al., 2021; Liu et al., 2022; Mattei et al., 2022). The fact that unique gene families in *E. vivipara* are significantly enriched in DNA methylation and demethylation processes (q‐value = 8.17e‐5), and regulation of histone H3K27 trimethylation (q‐value = 5.08e‐4) (Figure S2E; Table S9E), indicates that epigenetics may play an important role in gene regulation in this species. Further exploration of epigenetic data from submerged to terrestrial *E. vivipara* culms will yield deeper insights into the role of these mechanisms in the C_3_ to C_4_ transition. Although C_4_ photosynthesis evolved in *E. vivipara* after it diverged with *C. esculentus* and before the WGD event, the polyploidy, subgenome integration and optimization enabled it to recruit flexible subtypes of C_4_ (Figure 7A–D). The mixture of NAD‐ME and the PCK C_4_ pathway can robustly afford higher photosynthetic efficiency under a broad range of light regimes (Wang et al., 2014). This is reflected in the significant enrichment in response to light stimulus, light harvesting, and photosynthesis, which are enriched by both the *E. vivipara* expanded genes and the expressed genes that significant correlated with the terrestrial C_4_ culms (Figure S6). Further analysis on preferential retention of genes in the two subgenomes will also provide additional clues for the role of WGD in optimizing its C_4_ photosynthesis. Altogether, these findings from our transplanting experiments and transcriptome analyses offer novel perspectives on the genetic control of C_4_ photosynthesis and environmental acclimation in amphibious plants, which can contribute to crop improvement and breeding strategies.

## MATERIALS AND METHODS

### Plant materials and growth conditions

Fifty *E. vivipara* plants were acquired from a farm of Liwan, Guangzhou in Guangdong provinces, China. The plants were divided into two groups, with one subsequently cultivated underwater and the other in nutrient‐rich soil. All of these were grown in a controlled room environment with a constant temperature of 22°C, relative humidity 60%, and a 16 h light photoperiod for 2 months. The terrestrial culms were harvested, cleansed with 75% ethanol, dried with absorbent paper, and immediately cryopreserved in liquid nitrogen to extract genomic DNA.

### Analysis of culm anatomy

*E. vivipara* plants from submerged (0 d) and terrestrial environments (1, 5, 9, 13, 30, and 60 d after transplantation) were selected for phenotype and culm section comparisons. Culm sections, each 1 cm in length, were fixed in formalin aceto‐alcohol solution for 24 h at room temperature, then trimmed and placed in embedding frames. Following dehydration in escalating concentrations of alcohol and embedding in paraffin wax, the blocks were cooled to −20°C. The solidified wax blocks were then trimmed, and sections cut with a microtome were mounted on slides and heated in a 60°C oven to adhere the sections to the slides. Staining with safranin allowed for observation under a fluorescence upright microscope (BX63F; Olympus, Tokyo, Japan).

### Genome and transcriptome sequencing and genome size estimation

For DNB and PacBio sequencing, we extracted high‐quality DNA from *E. vivipara* culms using the cetyltrimethylammonium bromide (CTAB) method (Allen et al., 2006). The DNA was then divided into two portions: one for constructing short‐read sequencing libraries according to DNB protocol, and the other for PacBio single‐molecule real‐time (SMRT) sequencing library preparation. DNA was fragmented by ultrasound on Covaris E220 (Covaris, Brighton, UK), then selected to 300–500 bp using magnet beads size selection. The selected DNA fragments were then repaired to obtain a blunt end with T4 DNA polymerase, and modified at the 3′‐end to get a dATP sticky end. The dATP tailed adaptor was ligated to both ends of the DNA fragments with T4 DNA ligase. The ligation product was amplified by PCR, and circularized to get a single‐stranded circular (ssCir) library. The ssCir library was then amplified through rolling circle amplification to obtain DNA Nanoball. Then, it was loaded to flow cell, and sequenced by DNBSEQ Platform with a 150 bp paired‐end sequencing (PE150) strategy, generating approximately 88.25 Gb of data. For SMRT sequencing, we constructed the CLR type library in accordance with the PacBio guidelines, produced approximately 77.83 Gb of data with an average read length of about 26.11 kb and a subreads N50 length of approximately 31.56 kb.

For Hi–C sequencing, we optimized the construction method for *E. vivipara*'s Hi–C libraries based on existing protocols (Rao et al., 2014). Culms were cross‐linked under vacuum infiltration with 3% formaldehyde at 4°C for 30 min and quenched with 0.375 mol/L glycine for 5 min. After lysing the cross‐linked samples, endogenous nuclease activity was halted with 0.3% sodium dodecyl sulfate, and the chromatin‐DNA was digested using 100 U MboI restriction enzyme (NEB, Beijing), then labeled with biotin‐14‐dCTP (Invitrogen, Beijing), and ligated using 50 U T4 DNA ligase (NEB). Post‐cross‐link reversal, DNA was purified using the QIAamp DNA Mini Kit (Qiagen, Beijing) as per the manufacturer's instructions. The purified DNA was then sheared to 300–500 bp fragments as we described above in DNB sequencing, processed for blunt‐end repair, A‐tailing, adapter ligation, followed by biotin‐streptavidin‐mediated pull‐down, and PCR amplification. The Hi–C libraries were quantified and sequenced on the DNBSEQ Platform with PE150 strategy, yielding approximately 66.96 Gb of data.

For mRNA sequencing, *E. vivipara* culms from submerged (0 d) and terrestrial environments (1, 9, 13, 30, and 60 d after transplantation) were used for RNA extraction with the RNA Prep Pure Plant Kit (Tiangen, Beijing). RNA integrity was assessed using the Bioanalyzer 2100 system (Agilent Technologies, CA, USA). CDNA libraries were prepared following the DNB protocol, sequenced using the PE150 strategy, and produced approximately 129.43 Gb of data.

The genome size and heterozygosity ratio of *E. vivipara* were estimated using gce software (v1.0.0) based on k‐mer distribution with parameters set as −k 21 −a 0 −d 0 for the kmer_freq_hash step and −m 1 −b 1 for the gce step (Liu et al., 2013). The genome size calculation was performed using the formula: Genome size = k‐mer coverage/mean k‐mer depth.

### Genome assembly and chromosome sequence construction

PacBio CLR reads were assembled using Canu (v2.0) with parameters set to genomeSize = 1.05 g, minOverlapLength = 700, and minReadLength = 1,000 (Koren et al., 2017). Bubble sequences in the assembled contigs were removed using minimap2 (v2.17) with −x asm5 and purge_dups (v1.2.3) with −T 2 for further purification (Li, 2018; Guan et al., 2020). Short reads were then aligned back to the contigs using Bwa mem (v0.7.17) and corrections were made using Pilon (v1.24) with default settings (Li and Durbin, 2009; Walker et al., 2014). These refined contigs were organized into chromosome sequences with Hi–C data employing Juicer (v1.6) with −s MboI and 3D‐DNA (v190716) for scaffolding (Dudchenko et al., 2017). Manual adjustments and interaction intensity heatmaps were generated using Juicebox (v1.11.08) (Durand et al., 2016). Subphaser was utilized to assess the polyploidy level of the *E. vivipara* genome (Jia et al., 2022).

### Repeat and gene annotation

Extensive de‐novo TE Annotator (v1.9.6), RepeatModeler (v2.0.1), and RepeatMasker (v4.1.2, http://repeatmasker.org) were employed for repeat sequence identification in the *E. vivipara* genome (Ou et al., 2019; Flynn et al., 2020). Initially, EDTA was used to train the pseudomolecule and create transposon element library files with parameter –anno 1 –force 1 –debug 1 –sensitive 1 –evaluate 1. RepeatModeler was used to generate the *E. vivipara* repeat database file with default settings. These libraries were combined and used as input for RepeatMasker to annotate the genome's repeat sequences with –a –html –gff parameters.

The MAKER3 pipeline, which integrates transcriptome evidence, homologous proteins, and *ab initio* predictions, was used for gene structure annotation (Cantarel et al., 2008). For transcriptome evidence, the RNA‐seq reads were aligned to the reference genome by HISAT2 and the transcripts were assembled by StringTie and also *de novo* assembled by Trinity (Grabherr et al., 2011; Pertea et al., 2015; Kim et al., 2019). For homology‐based prediction, we used the non‐redundant plant homologous protein sequences in the UniProt Swiss‐Prot database (www.uniprot.org/downloads) and published protein sequences from *Oryza sativa*, *Zea mays*, *Arabidopsis thaliana*, and the closely related species *Carex littledalei* (Ouyang et al., 2007; Lamesch et al., 2012; Can et al., 2020; Hufford et al., 2021). The transcriptome evidence and homologous proteins were passed to MAKER3 for the first round to deduce preliminary gene structures. The *ab initio* predictions were first generated with the BRAKER3 pipeline including GeneMark‐ETP and AUGUSTUS (Stanke et al., 2006; Brůna et al., 2021; Gabriel et al., 2024), with integrated transcript and protein sequences from *Carex littledalei* as evidence. The Mikado pipeline was selected for the best transcripts assembled with Trinity, StringTie, Cufflinks, Strawberry, and Class2 (Trapnell et al., 2010; Song et al., 2016; Liu and Dickerson, 2017; Mapleson et al., 2018; Venturini et al., 2018). Transcripts were used for program to assemble spliced alignments (PASA) with two rounds to refine the gene structures (Haas et al., 2003). Finally, the BRAKER3 and PASA results, as well as the predicted gene models from the first round of MAKER3, were used as input files for the second round of MAKER3 to generate the final gene annotations.

For gene functional annotation, the longest representative protein sequence of each gene was used as a query for Diamond BLAST against the NR and Swissprot databases to identify homologous proteins with –e 1e‐5 –sensitive –max‐target‐seqs. 1 –p 20 parameters (Buchfink et al., 2021). Functional domains and potential GO terms within protein sequences were identified using GO, KEGG, and Pfam databases via the eggNOG‐mapper v2 online tool (Cantalapiedra et al., 2021).

### Ortholog clustering and gene family analysis

The nuclear protein‐coding genes of the other 12 species, including seven sedges, one Brassicaceae, and two species each from Juncaceae and Poaceae, were downloaded according to the published article (Ouyang et al., 2007; Lamesch et al., 2012; Can et al., 2020; Hufford et al., 2021; Hofstatter et al., 2022; Planta et al., 2022; Qu et al., 2022; Ning et al., 2023; Zhao et al., 2023). The representative longest protein per locus was selected and clustered using Orthofinder2, employing parameters –M msa –A mafft –T fasttree –a 40 –t 40 –S diamond (Emms and Kelly, 2019). The jvenn online tool facilitated the comparison and visualization of unique gene families in *E. vivipara* relative to other species (Bardou et al., 2014). CAFE5 was used to calculate the expansion and contraction of gene families across each lineage with *P*‐value < 0.01 as the threshold for significant expansion or contraction (Mendes et al., 2021). GO enrichment for genes in unique, expanded, and contracted gene families was performed using the SEA method in AgriGO v2 (Tian et al., 2017). Synonymous substitution rates (*K*s values) for homologous gene pairs across these species were calculated using *K*a*K*s_Calculator2.0 (Wei et al., 2010).

### Phylogenetic inference

The species tree was constructed with 756 single‐copy gene families with a minimum of nine species present. Phylogenetic inference and divergence times within the phylogeny were estimated with Beast (v2.7.6) (Bouckaert et al., 2019). The Strict Clock Model was employed, with two referenced divergence times for the genera *Juncus* and *Zea* (CI: 77.70–111.00 Mya), the genera *Juncus* and *Rhynchospora* (CI: 65.60—88.00 Mya), referencing Timetree5 (Bremer, 2002; Kumar et al., 2017; Kumar et al., 2018; Li et al., 2019). The Gamma Site model was used as the tree likelihood site model for leaves, and the Yule model as the tree prior. Markov chain Monte Carlo analyses were run for 10,000,000 generations. The resulting trees were summarized using TreeAnnotator v2.7.6 in Beast with 50% as burn‐in, to obtain a maximum clade credibility tree with mean node ages.

### Gene synteny and cross‐species chromosome comparisons

Paralogous genes within the *E. vivipara* genome were identified with MCScanX, and their synteny visualized using Circos (Krzywinski et al., 2009; Wang et al., 2012). The genomes of *E. vivipara*, *C. esculentus*, *C. littledalei*, *R. pubera*, *R. tenuis*, and *R. breviuscula* were firstly aligned to pinpoint synteny regions using Mummer4 with –L 100 as the parameter (Marçais et al., 2018), the alignment length for their homologous chromosomes were filtered, and their synteny was displayed by NGenomeSyn (He et al., 2023).

For *J. effusus*, which possesses monocentromeres, its genome and annotation files were retrieved from public datasets (Planta et al., 2022). Its Hi–C interaction pattern was generated using Juicer, 3D‐DNA, and Juicebox, following the same protocol as for the *E. vivipara* genome. The density of genes, mRNA‐seq data, satellite DNA, *Helitron*, *Copia*, and *Gypsy* were quantified with a window size of 150 kb for *J. effusus*, and 1 Mb for *E. vivipara*. Density patterns were subsequently compared using integrative genomics viewer (Robinson et al., 2011).

### Transcriptome and positive selection analysis

RNA‐seq data were aligned to the reference genome using Hisat2 (v2.2.1) (Kim et al., 2019), with gene expression quantified by StringTie (v1.3.5) (Pertea et al., 2015). Genes exhibiting expression levels of FPKM >1.0 were considered expressed and included in subsequent analyses. WGCNA and TBtools were used to analyze and show the gene expression patterns for the six stages of culms (Langfelder and Horvath, 2008; Chen et al., 2020). The gene expression was first normalized and the genes with FPKM < 1 in more than 90% of samples were removed, then 6,000 genes with the best MAD values were filtered for further analysis. Cytoscape was further used to display the gene co‐expression networks (Smoot et al., 2011). Differential expression between the culms of two states was determined using DESeq2, employing thresholds of |fold‐change| > 2, FPKM > 1, and q‐value < 0.05 to identify DEGs (Love et al., 2014). Fold‐change values were derived from normalized read counts, a method noted for its enhanced accuracy over FPKM‐based calculations (Zhao et al., 2021). Genes involved in C_4_ pathway in subgenomes A and B were further identified and analyzed based on the photosynthesis‐related expression patterns: lower expression in the aquatic C_3_ state and higher expression in the terrestrial C_4_ state. The amino acids that under positive selection in C_4_ PEPC genes evolution across Cyperaceae, Juncaceae, Poaceae, and Brassicaceae families were calculated with phylogenetic analysis by maximum likelihood (PAML) (Yang, 2007).

### Reverse transcription – qPCR

Reverse transcription qPCR was performed to validate the relative expression of the mRNA‐seq results, by selecting 11 genes, including nine DEGs and two non‐DEGs between the transcriptome of 0 d versus 13 d and 0 d versus 30 d. The actin gene (*ELviv04G0163450*) was used as internal control to normalize relative expression. The reverse transcription and RT‐qPCR were performed according to previously described protocols (Li et al., 2022). The primer sequences are listed in Table S11.

## CONFLICTS OF INTEREST

The authors declare no conflict of interest.

## AUTHOR CONTRIBUTIONS

S.C., H.L., and M.S. designed the project. H.L., Y. Zhang, and W.N. collected the material and sequenced the data. H.L., Y. Zhang, Z.W., Y. Zuo, and X.Z. conducted the experiments. H.L., H.Z., Y. Zhang, X.L., Y. Zuo and W.W. analyzed the data. H.L. and Y. Zuo led the manuscript writing and M.S. led the revision. S.C. and M.S. agreed to serve as corresponding authors. All authors contributed to manuscript revision and approved of the contents of this paper.

## Supporting information

Additional Supporting Information may be found online in the supporting information tab for this article: http://onlinelibrary.wiley.com/doi/10.1111/jipb.13765/suppinfo

**Figure S1.** The K‐mer distribution, BUSCO evaluation, and genes functional annotation for the genome of *Eleocharis vivipara*
**Figure S2.** GO enrichment analysis of genes in expanded, significantly expanded, contracted, significantly contracted, and unique gene families in *E. vivipara genome*
**Figure S3.** Hi‐C interaction intensity map for the 21 chromosomes of *Juncus effusus*
**Figure S4.** The three‐dimensional principal component analysis (3d‐PCA) and weighted gene co‐expression network analysis (WGCNA) results for the mRNA‐seq of the *E. vivipara* culms in six different stages
**Figure S5.** Genes co‐expression networks enriched in photosynthesis related terms in terrestrial culms
**Figure S6.** The overlap relationship between the terrestrial culms enriched genes and expanded gene families enriched genes that related to photosynthesis in *E. vivipara*
**Figure S7.** Plant morphology of the terrestrial (left) and submerged type (right) for *E. vivipara*
**Figure S8.** The validation of mRNA‐seq results by RT‐qPCR
**Figure S9.** The top 25 of GO enrichment in BP for up‐ and down‐DEGs
**Figure S10.** Genes expression and functional analysis of up‐DEGs enriched in water deprivation, response to abscisic acid, and down‐DEGs enriched in transcription factor (TF) activity in terrestrial culms of *E. vivipara*
**Figure S11.** The phylogeny and selection pressure of PEPC protein in the thirteen species
**Figure S12.** Comparison of C4 core genes’ expression level between submerged (0 d) and terrestrial (30 d) culms of *E. vivipara*
**Table S1.** Summary of sequenced data for *E. vivipara* genome assembly
**Table S2.** Quality values of the assembled contig‐level genome of *E. vivipara*
**Table S3.** The completeness evaluation of the contig‐level *E. vivipara* genome based on BUSCO database, mapping ratio of DNB short reads, and transcriptome data
**Table S4.** Detail of the ten long chromosomes constructed with Hi‐C interaction intensity map
**Table S5.** The content of repeat sequence in *E. vivipara* chromosome‐level genome
**Table S6.** Features of the assembly and protein‐coding genes for the chromosome‐level genome of *E. vivipara*
**Table S7.** Function annotation of *E. vivipara* genes based on the five databases
**Table S8.** Comparison of gene families for the thirteen species in phylogenetic tree
**Table S9.** Gene ontology (GO) enrichment of genes in expanded (A), significant expanded (B), contracted (C), significant contracted (D), and unique (E) gene families of *E. vivipara* genome
**Table S10.** Summary of mRNA‐seq data and numbers of expressed genes in each replicate
**Table S11.** List of primer sequences for selected genes used to perform the RT‐qPCR experiment
**Table S12.** GO enrichment of genes in 2,602 up‐DEGs (A) and 1,095 down‐DEGs (B)
**Table S13.** List of genes involved in C4 core pathway used to compare the contribution of subgenome A and B in *E. vivipara*

## Data Availability

The sequencing data and genome assembly presented in this study are accessible in the Genome Warehouse (GWH) at the National Genomics Data Center (NGDC) under BioProject accession number PRJCA021868. Comprehensive genome sequencing datasets, including PacBio long‐reads, Hi–C data, and DNB short reads for both DNA and RNA sequencing, have been deposited in the Genome Sequence Archive (GSA) of NGDC under accession number CRA013818. The assembled genome is available in the GWH of NGDC with accession number GWHEQUP00000000. Annotations regarding repeat sequences, gene structures, and functional predictions are also hosted on the Figshare database (https://doi.org/10.6084/m9.figshare.24902079.v2).
