# Supplementary material for: The genome of Eleocharis vivipara elucidates the genetics of C3–C4 photosynthetic plasticity and karyotype evolution in the Cyperaceae
Source: J Integr Plant Biol. 2024 Aug 23;66(11):2505–27. doi: 10.1111/jipb.13765 (PMC11583847; doi:10.1111/jipb.13765)
Supplement: Supplementary file 1 — Figure S1. The K‐mer distribution, BUSCO evaluation, and genes functional annotation for the genome of Eleocharis vivipara Figure S2. GO enrichment analysis of genes in expanded, significantly expanded, contracted, significantly contracted, and unique gene families in E. vivipara genome Figure S3. Hi‐C interaction intensity map for the 21 chromosomes of Juncus effusus Figure S4. The three‐dimensional principal component analysis (3d‐PCA) and weighted gene co‐expression network analysis (WGCNA) results for the mRNA‐seq of the E. vivipara culms in six different stages Figure S5. Genes co‐expression networks enriched in photosynthesis related terms in terrestrial culms Figure S6. The overlap relationship between the terrestrial culms enriched genes and expanded gene families enriched genes that related to photosynthesis in E. vivipara Figure S7. Plant morphology of the terrestrial (left) and submerged type (right) for E. vivipara Figure S8. The validation of mRNA‐seq results by RT‐qPCR Figure S9. The top 25 of GO enrichment in BP for up‐ and down‐DEGs Figure S10. Genes expression and functional analysis of up‐DEGs enriched in water deprivation, response to abscisic acid, and down‐DEGs enriched in transcription factor (TF) activity in terrestrial culms of E. vivipara Figure S11. The phylogeny and selection pressure of PEPC protein in the thirteen species Figure S12. Comparison of C4 core genes’ expression level between submerged (0 d) and terrestrial (30 d) culms of E. vivipara Table S1. Summary of sequenced data for E. vivipara genome assembly Table S2. Quality values of the assembled contig‐level genome of E. vivipara Table S3. The completeness evaluation of the contig‐level E. vivipara genome based on BUSCO database, mapping ratio of DNB short reads, and transcriptome data Table S4. Detail of the ten long chromosomes constructed with Hi‐C interaction intensity map Table S5. The content of repeat sequence in E. vivipara chromosome‐level genome Table S6. Features of th [file JIPB-66-2505-s001.docx]

**Supplemental Figures and Tables for:**

**Liu *et al.,* The Genome of *Eleocharis vivipara* Elucidates the Genetics of C_3_-C_4_ Photosynthetic Plasticity and Karyotype Evolution in the Cyperaceae**

**Content**

**Supplemental Figures ..............................................................................................................3**

*Figure S1....................................................................................................................................3*

*Figure S2....................................................................................................................................4*

*Figure S3....................................................................................................................................7*

*Figure S4....................................................................................................................................8*

*Figure S5....................................................................................................................................9*

*Figure S6..................................................................................................................................10*

*Figure S7..................................................................................................................................11*

*Figure S8..................................................................................................................................12*

*Figure S9..................................................................................................................................13*

*Figure S10................................................................................................................................14*

*Figure S11................................................................................................................................17*

*Figure S12................................................................................................................................18*

**Supplemental Tables ..............................................................................................................19**

*Table S1...................................................................................................................................19*

*Table S2...................................................................................................................................20*

*Table S3...................................................................................................................................21*

*Table S4...................................................................................................................................22*

*Table S5...................................................................................................................................23*

*Table S6...................................................................................................................................26*

*Table S7...................................................................................................................................27*

*Table S8...................................................................................................................................28*

*Table S9...................................................................................................................................29*

*Table S10.................................................................................................................................36*

*Table S11.................................................................................................................................37*

*Table S12.................................................................................................................................38*

*Table S13.................................................................................................................................44*

_
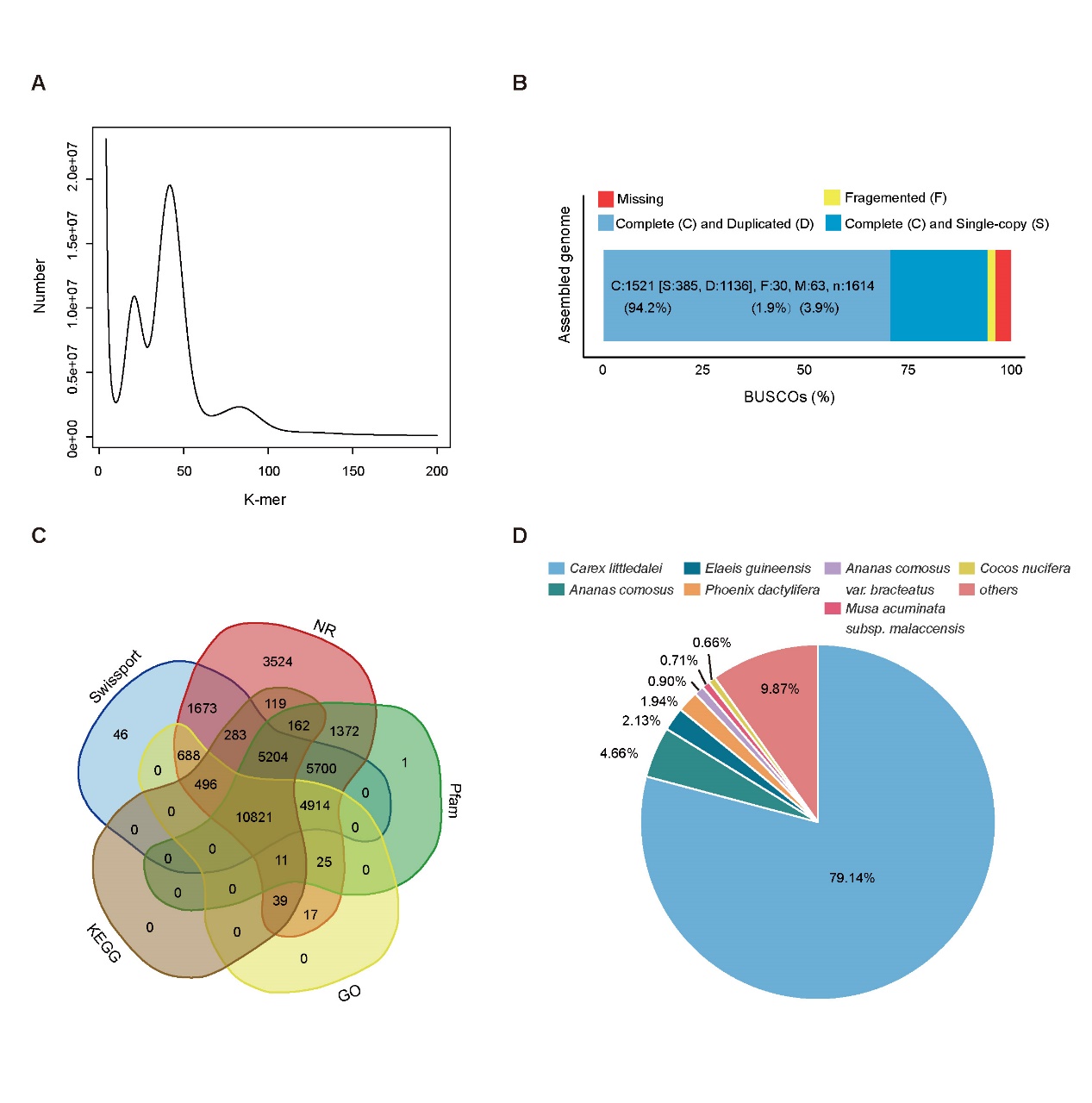
_

**Figure S1. The K-mer distribution, BUSCO evaluation, and genes functional annotation for the genome of *Eleocharis vivipara*.**

A. The genome size of it was estimated to be ~1.05 Gb based on the 21-mer distribution curve. B. Approximately 96.10% of the 1,614 BUSCO genes were annotated, and ~94.23% of them were complete. C. Venn diagram of annotated genes in NR, Pfam, GO, KEGG, and Swissport databases, with 35,094 (~90.52%) of the 38,769 genes could be annotated. D. The identified homologous genes of *E. vivipara* against the NR database using blastp, with *Carex littledalei* and *Ananas comosus* genes accounting for ~79.14% and ~4.66% of the total, respectively.


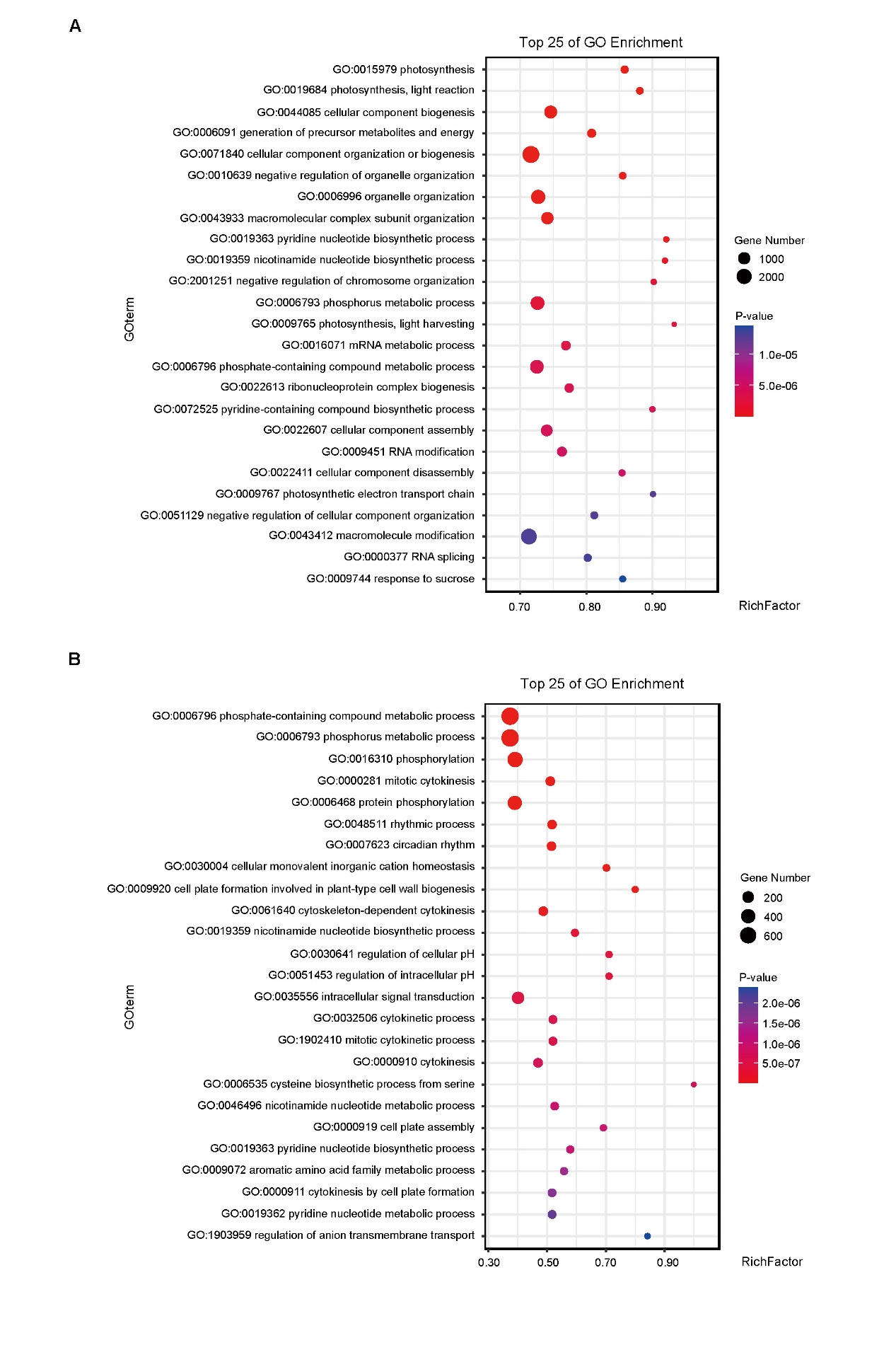


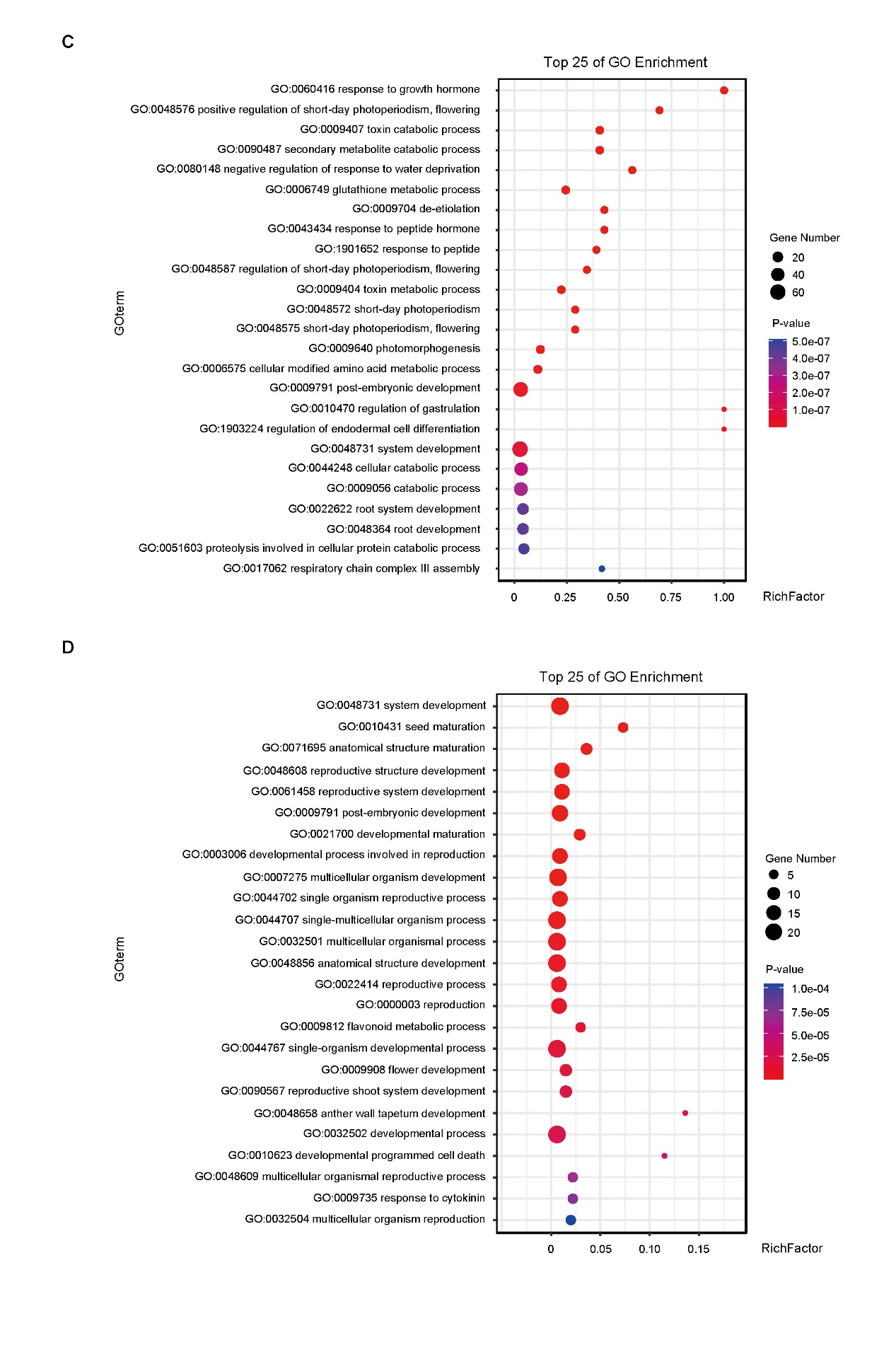


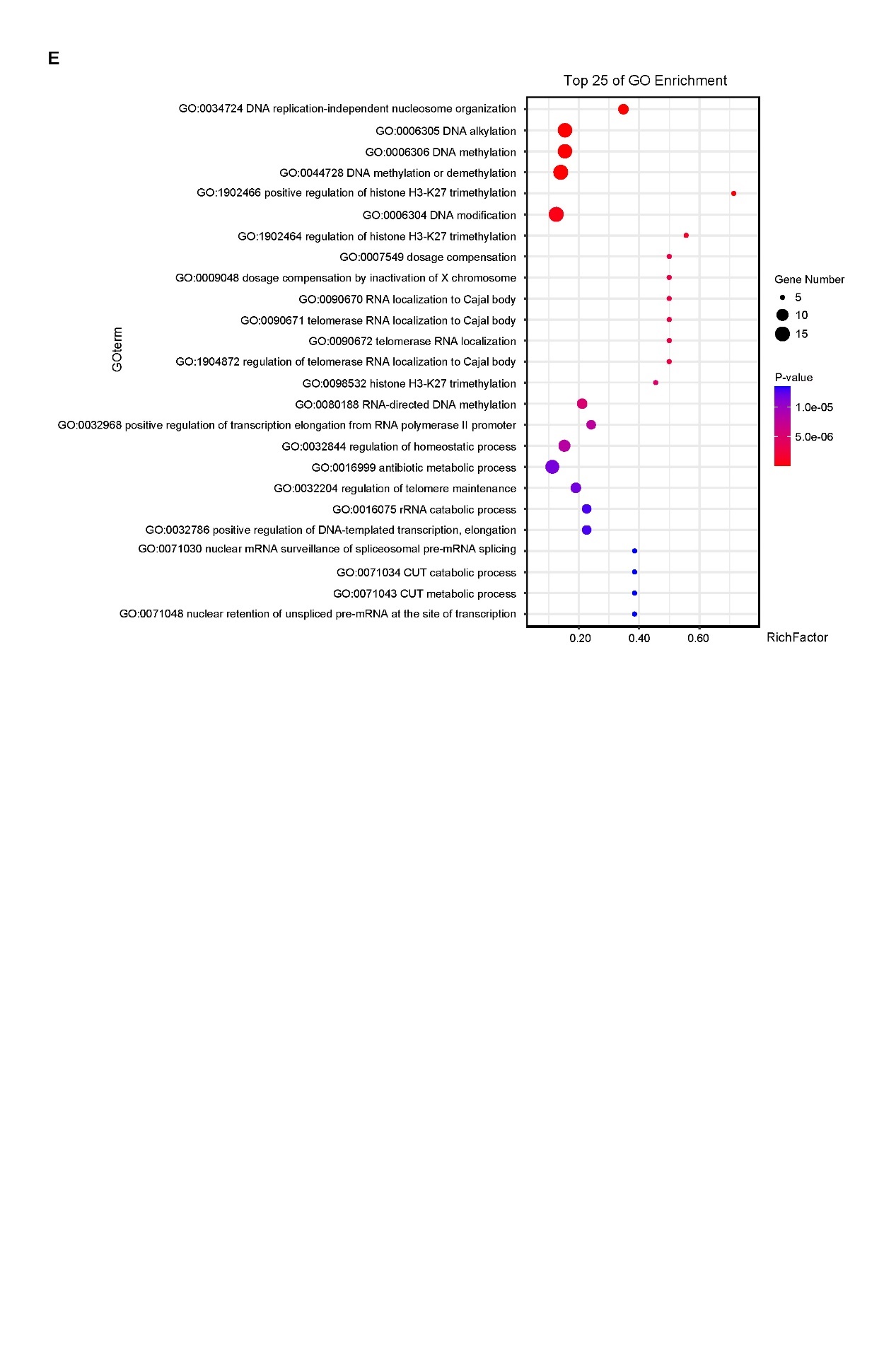


**Figure S2. GO enrichment analysis of genes in expanded, significantly expanded, contracted, significantly contracted, and unique gene families in *E. vivipara* genome.**

GO enrichment analysis of genes in expanded (A), significantly expanded (p-value < 0.01, B), contracted (C), significantly contracted (p-value < 0.01, D), and unique gene families (E) in *E. vivipara* genome. The top 25 of GO enrichment in biological process (BP) were displayed.


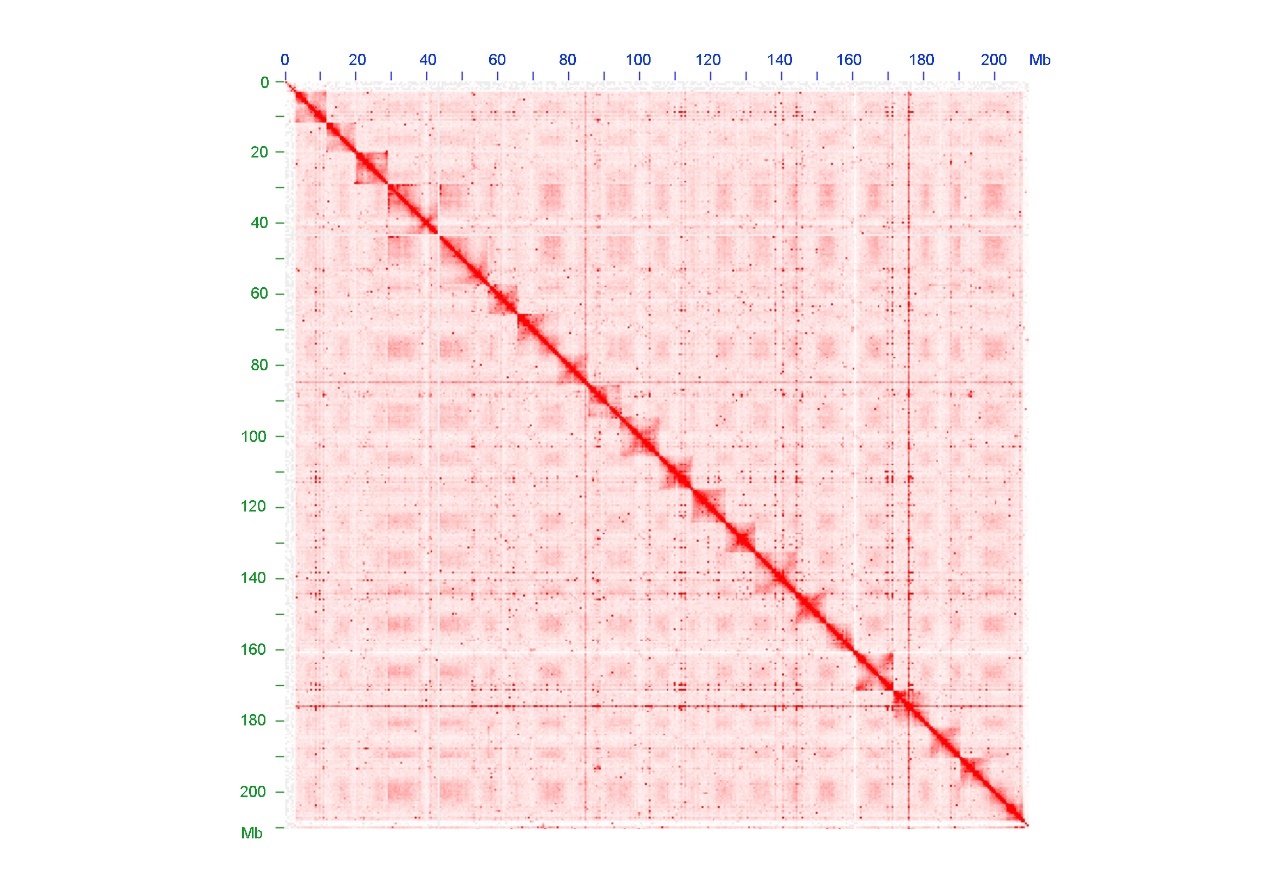


**Figure S3. Hi-C interaction intensity map for the 21 chromosomes of *Juncus effusus*.**

The pattern of its single chromosomes showed extensive contact in telomeres-to-centromere axis, which formed an obvious X-shape pattern and different from that of *E. vivipara* which showed uniform distribution.


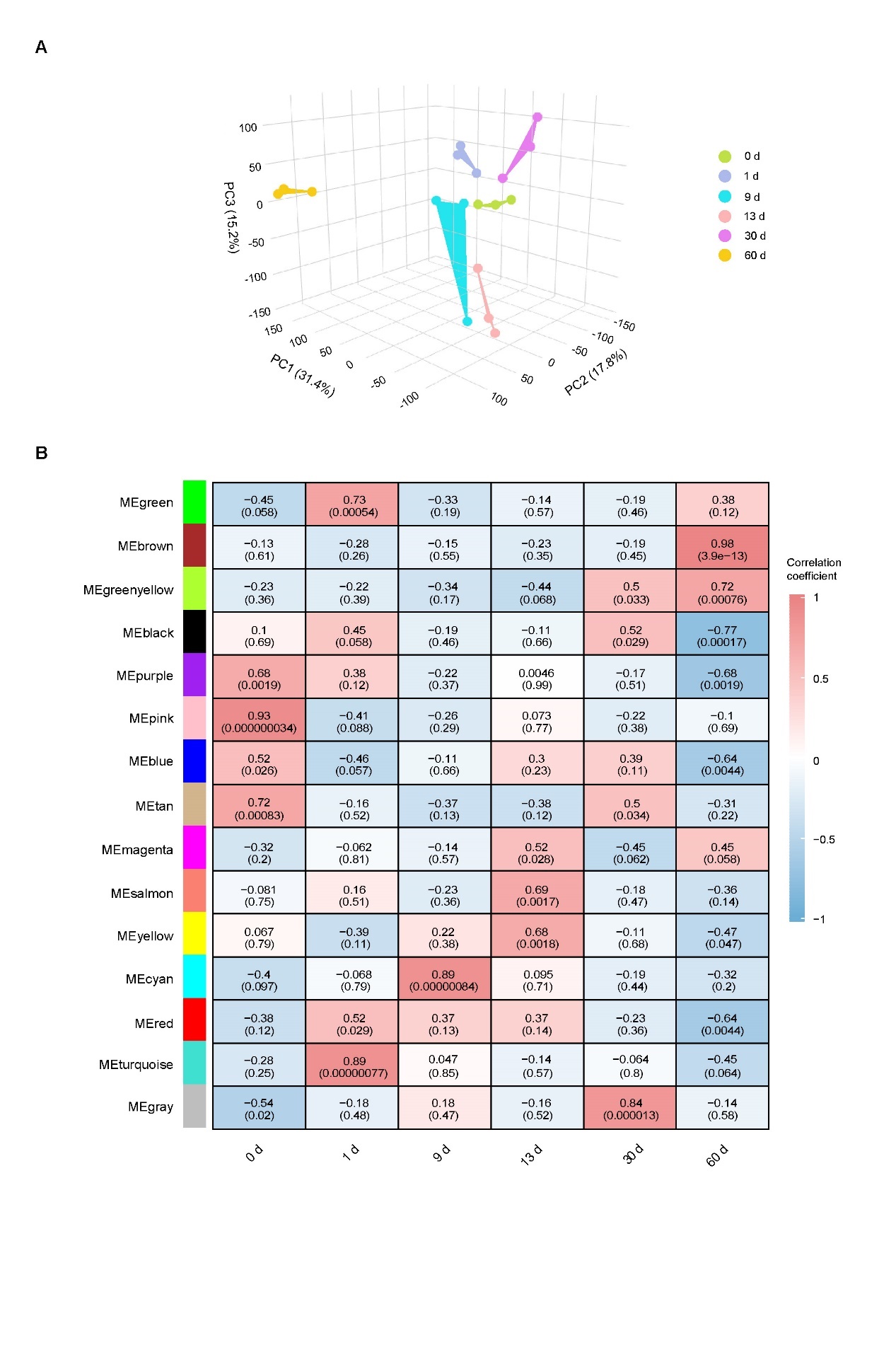


**Figure S4. The three-dimensional principal component analysis (3d-PCA) and** **weighted gene co-expression network analysis (WGCNA) results for the mRNA-seq of the *E. vivipara* culms in six different stages.**

A. PCA result displayed the PC1, PC2, and PC3 explained 31.4%, 17.8%, and 15.2% of the variation, respectively. B. The WGCNA results displayed these genes were divided into 15 modules. In each module, both the correlation coefficient (upper) and p-value (lower) for the genes expression and the stages were displayed.


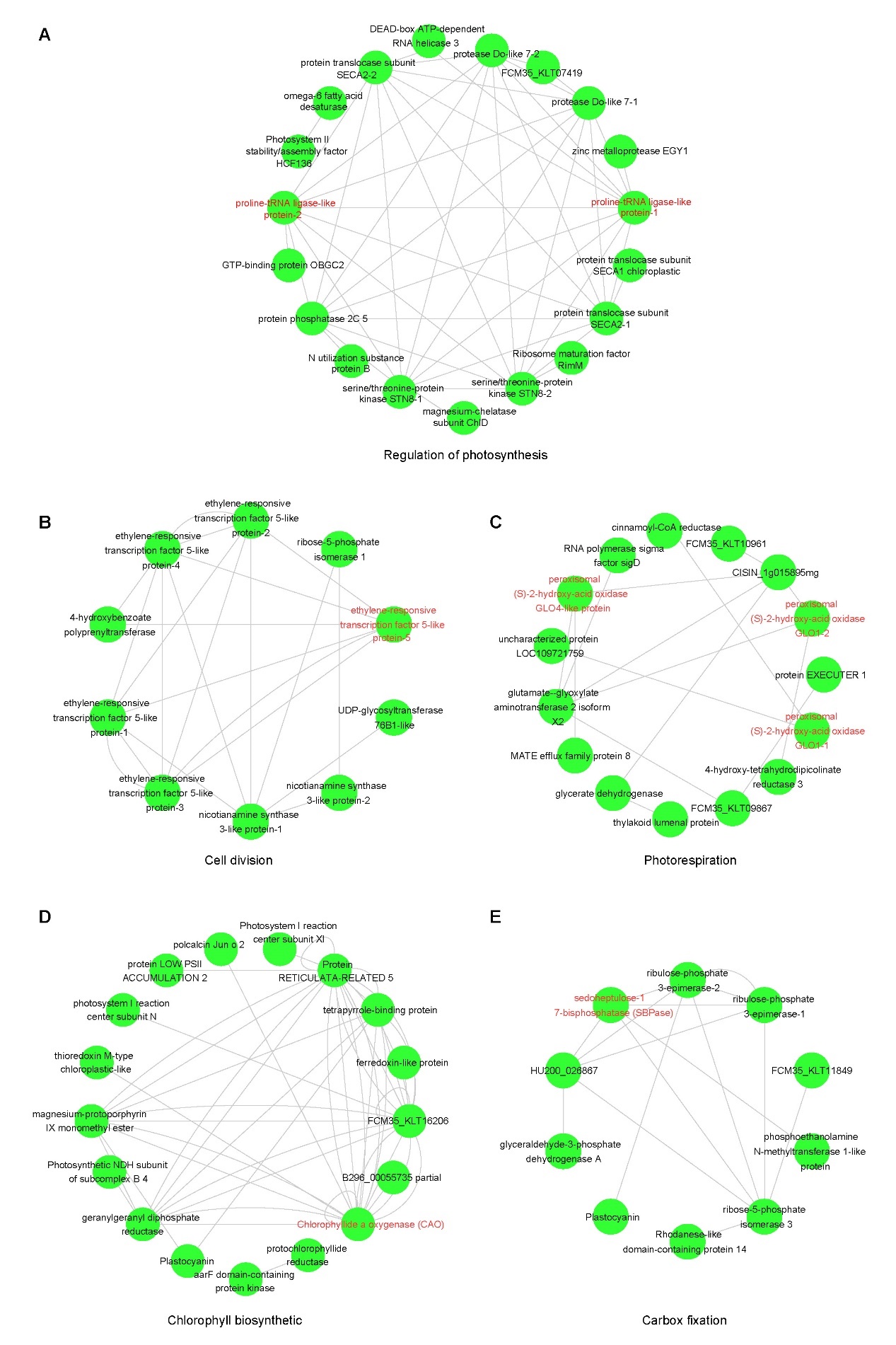


**Figure S5. Genes co-expression networks enriched in photosynthesis related terms in terrestrial culms**.

Here the representative and key hub genes in each network are highlighted in red font.


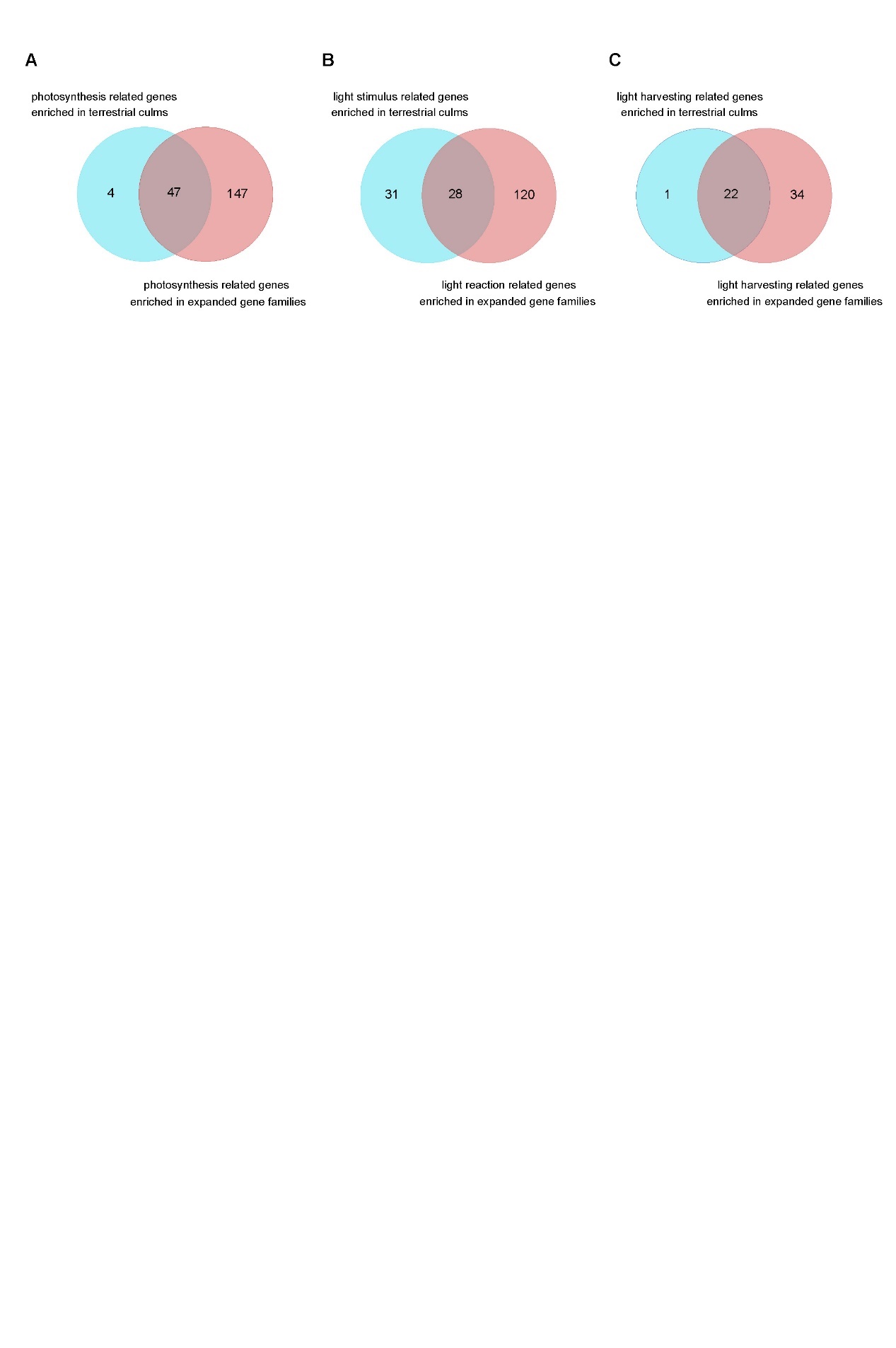


**Figure S6. The overlap relationship between the terrestrial culms enriched genes and expanded gene families enriched genes that related to photosynthesis in *E. vivipara***.

With 92.16% of photosynthesis related genes (A), 47.46% of the light stimulus related genes (B), and 95.65% light harvesting related genes (C) that enriched in terrestrial culm (brown module) could be found in corresponding terms of expanded gene families, respectively.

**
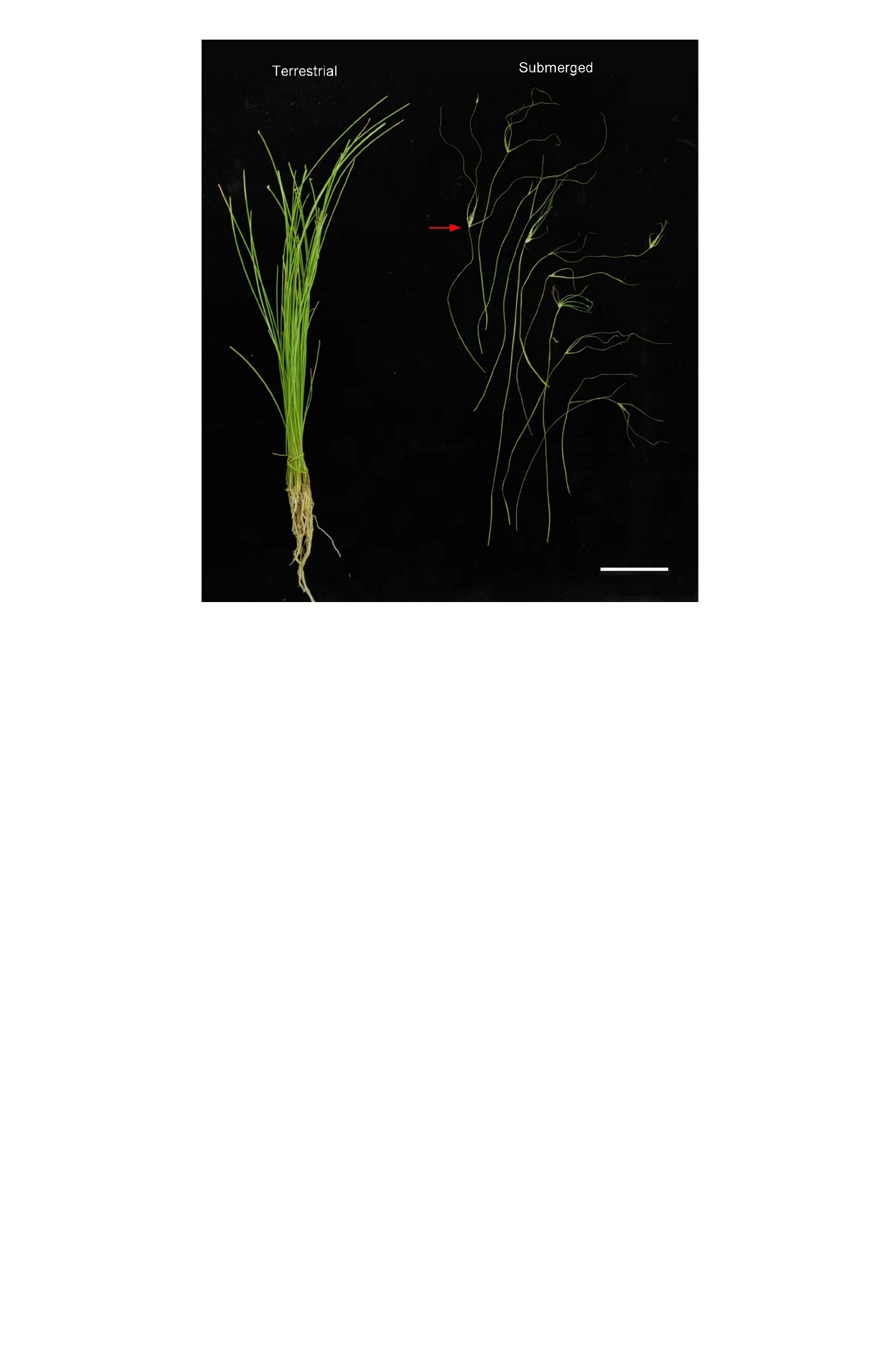
**

**Figure S7. Plant morphology of the terrestrial (left) and submerged type (right) for *E. vivipara.***

The red arrow indicated sterile spikelets. Bar = 3 cm.

**
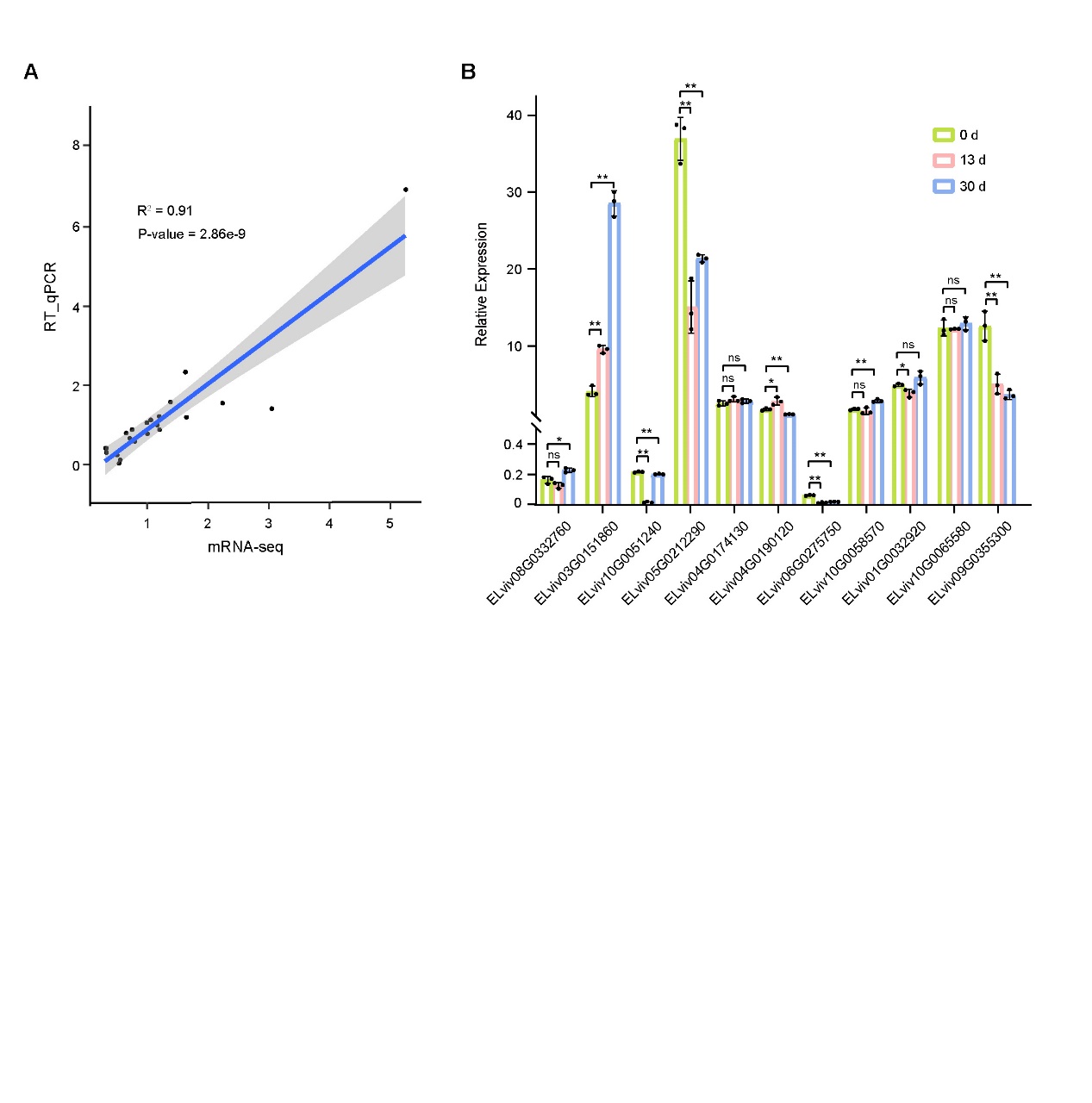
**

**Figure S8. The validation of mRNA-seq results by RT-qPCR.**

A. The gene expression correlation between mRNA-seq and RT-qPCR is 0.91. B. The validation of 9 DEGs and 2 non-DEGs identified between 0 d versus 13 d and 0 d versus 30 d by RT-qPCR. *, p-value < 0.05; **, p-value < 0.01.


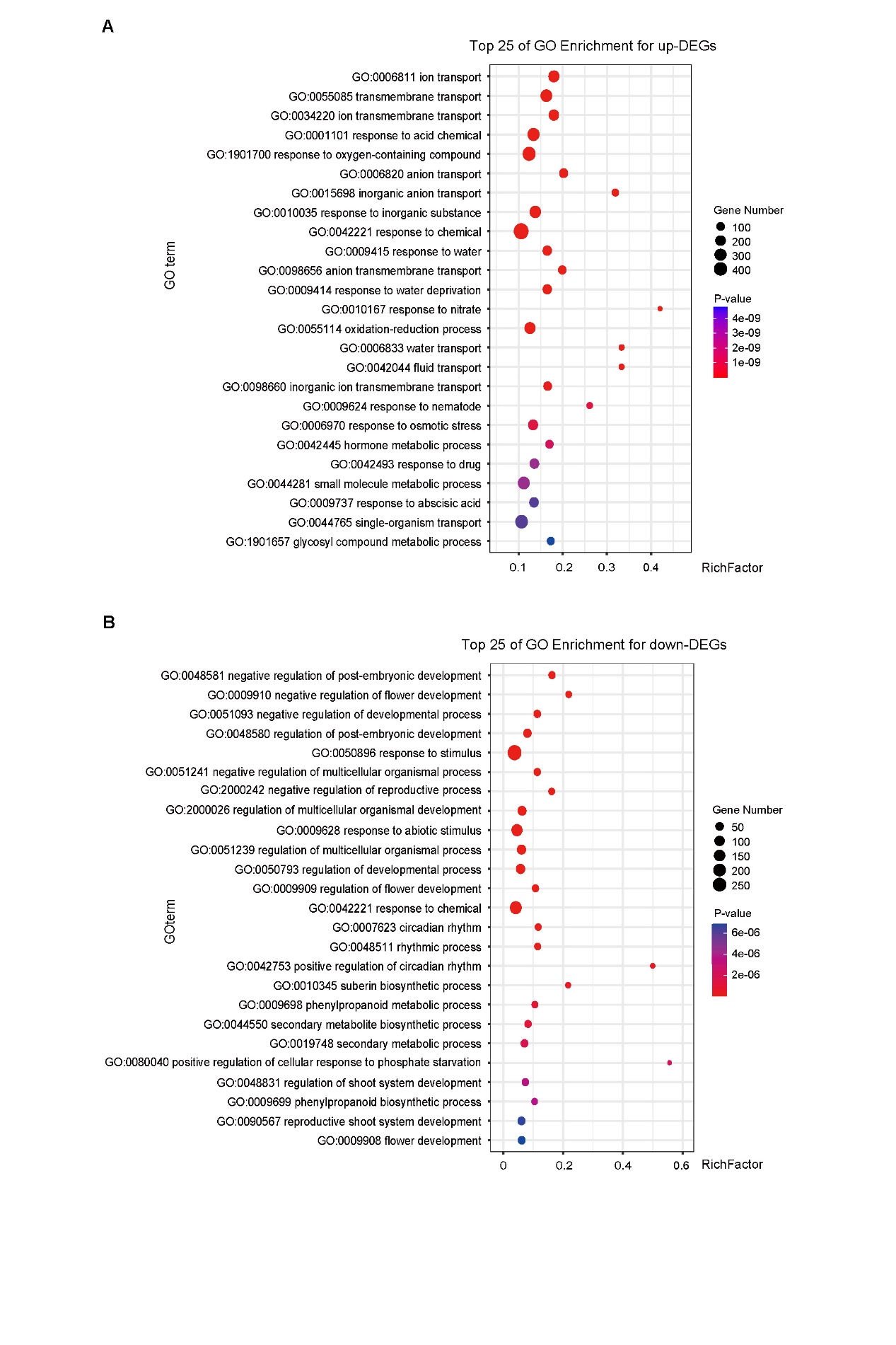


**Figure S9. The top 25 of GO enrichment in** **BP for up- and down-DEGs**.

A. Up-regulated differential expression genes. B. Down-regulated differential expression genes.

**
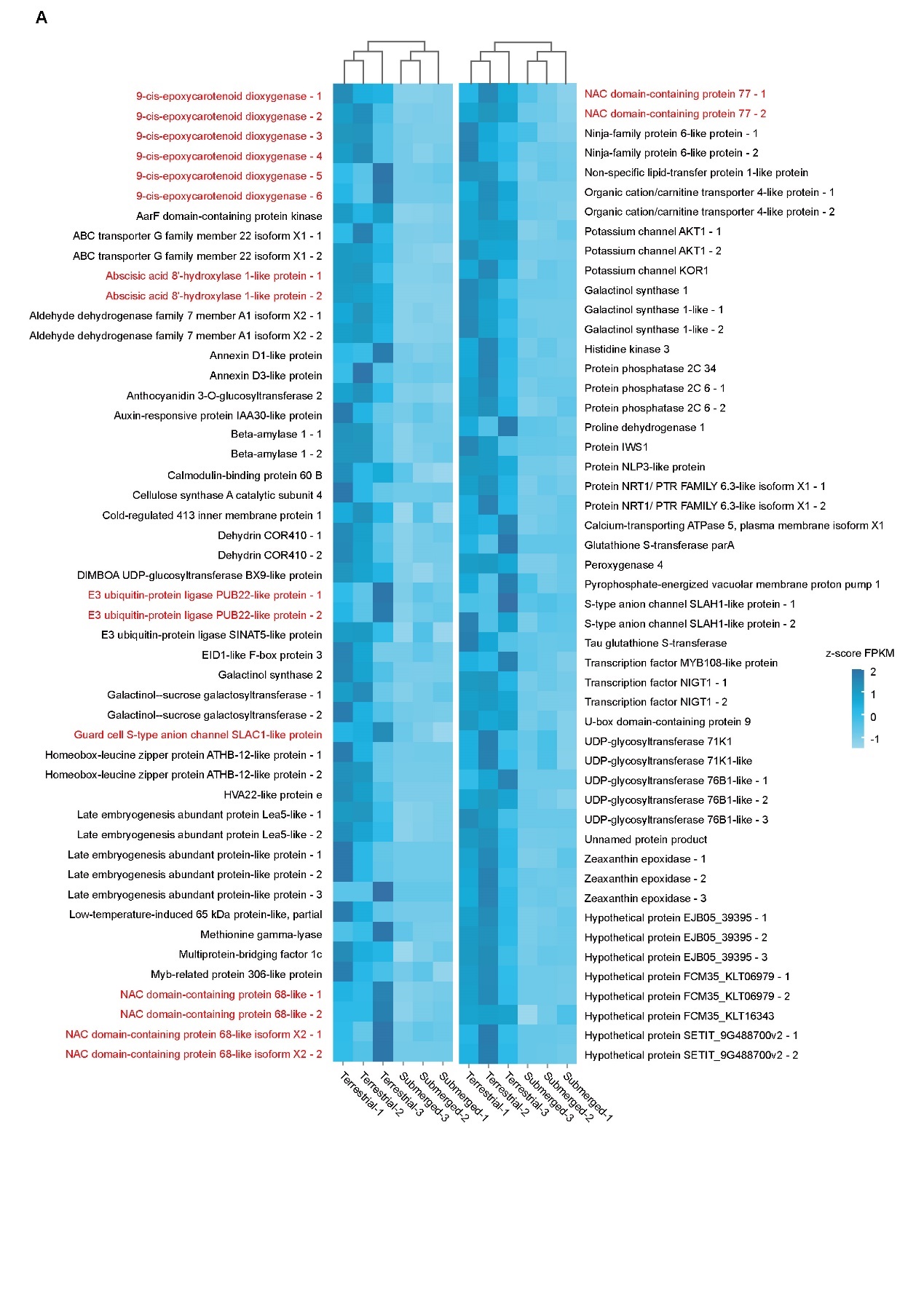
**

**
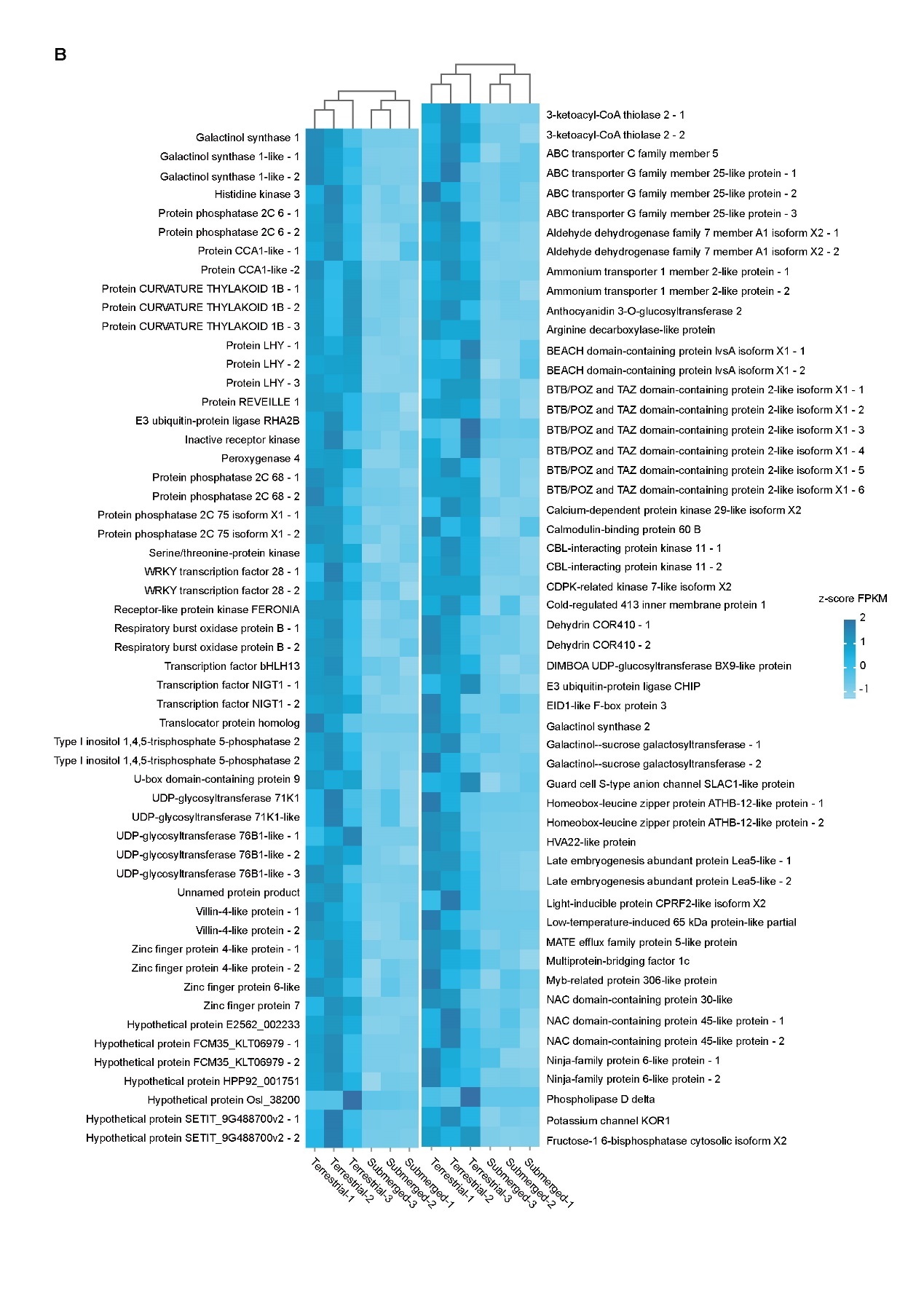
**

**
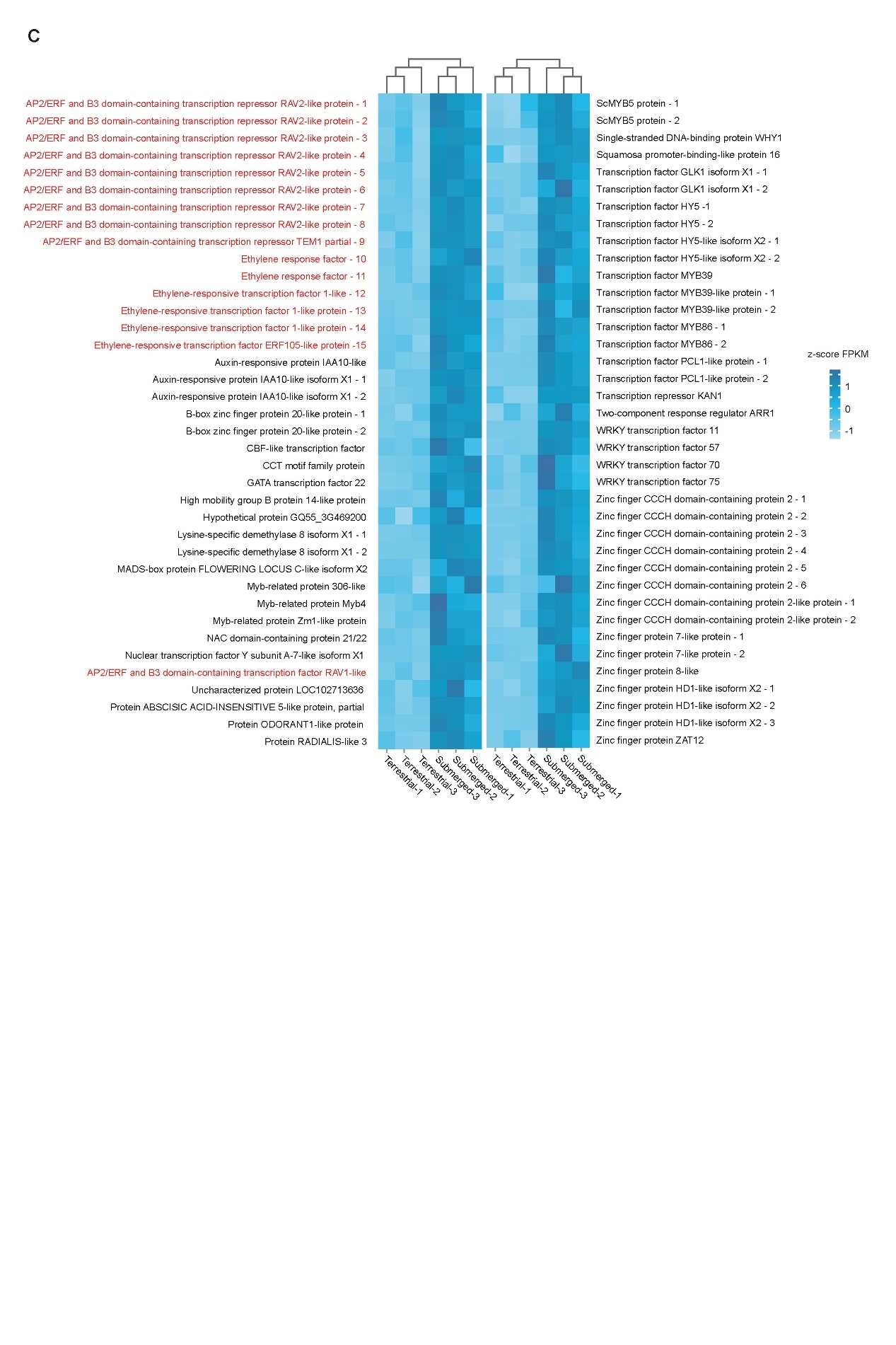
**

**Figure S10. Genes expression and functional analysis of up-DEGs enriched in water deprivation, response to abscisic acid, and down-DEGs enriched in transcription factor (TF) activity in terrestrial culms of *E. vivipara*.**

Genes expression and functional analysis of the 98 up-DEGs enriched in water deprivation (A), 107 up-DEGs enriched in response to abscisic acid (B), and 76 down-DEGs enriched in transcription factor (TF) activity (C) in terrestrial culms of *E. vivipara*.

**
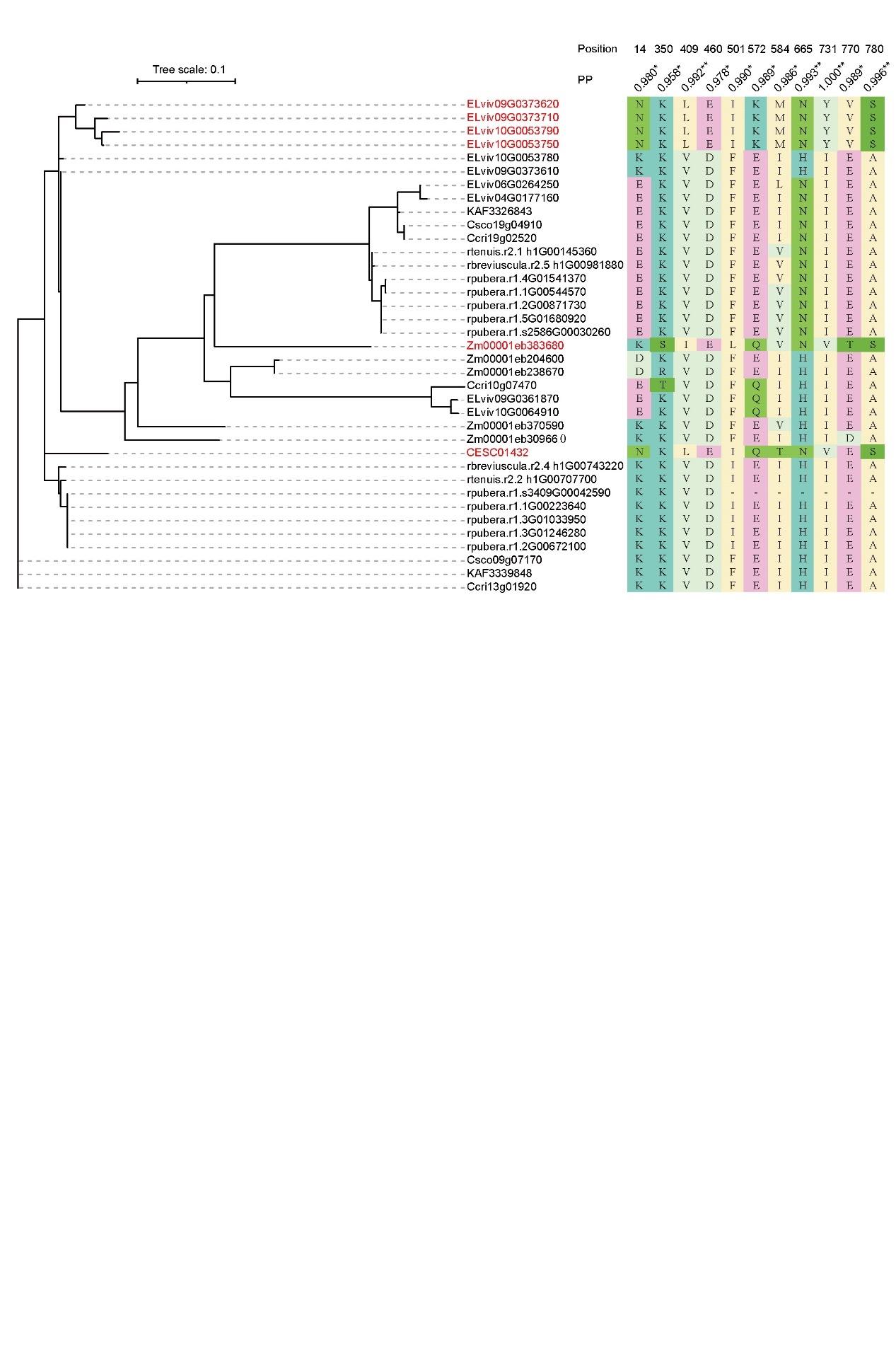
**

**Figure S11. The phylogeny and selection pressure of PEPC protein in the thirteen species.** The gene names which display a serine at position 780 in the C-terminal end of C_4_ PEPC protein in maize are highlighted with red font. PP, posterior probability. *, PP > 0.95; **, PP > 0.99.

**
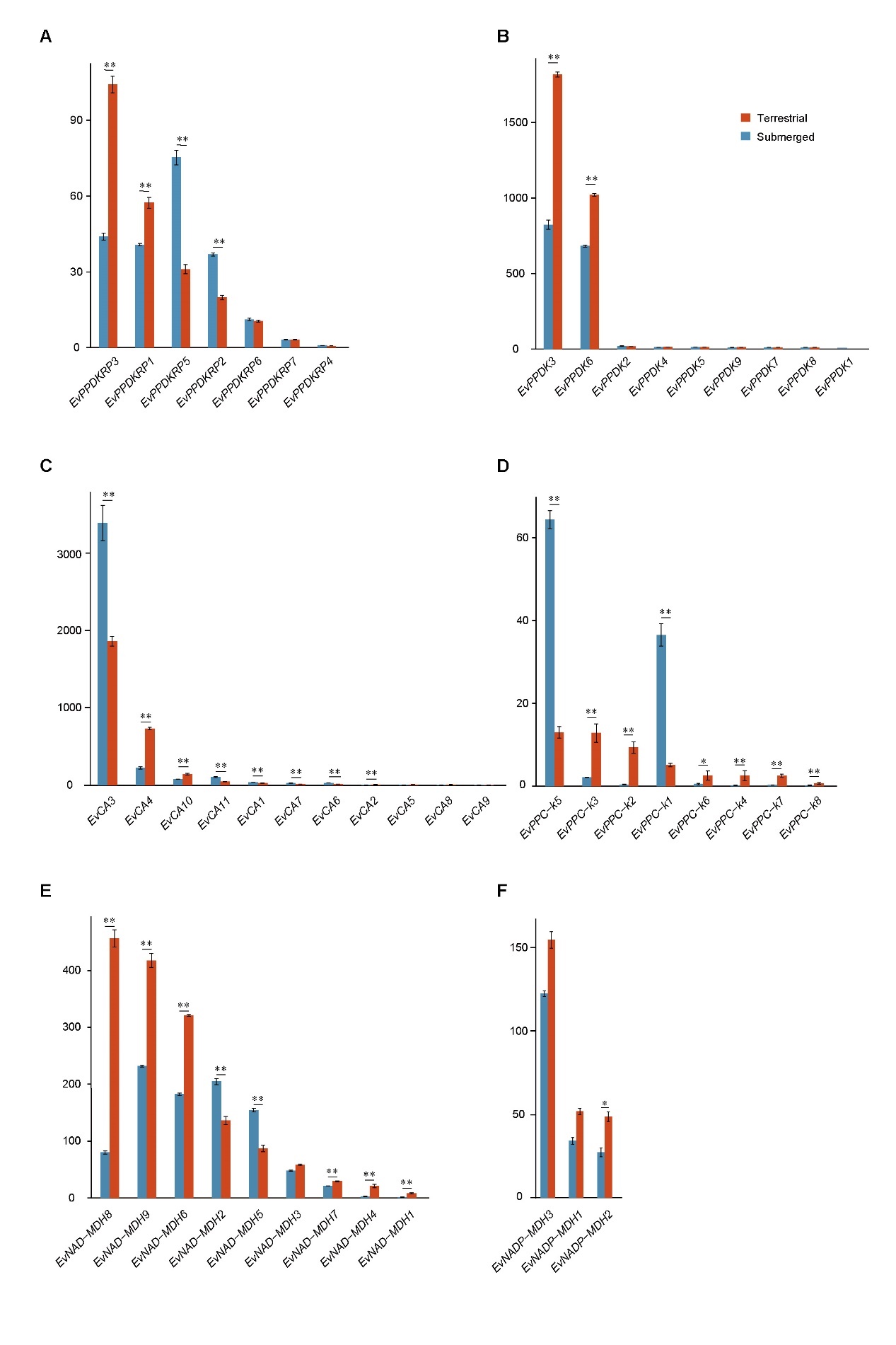
**

**Figure S12. Comparison of C_4_ core genes’ expression level between submerged (0 d) and terrestrial (30 d) culms of *E. vivipara***.

*, q-value < 0.05; **, q-value < 0.01.

**Table S1. Summary of sequenced data for *E. vivipara* genome assembly.**

| Platform | DNB | PacBio | Hi-C |
| --- | --- | --- | --- |
| Reads number (M) | 588.33 | 2.98 | 464.40 |
| Data volume (Gb) | 88.25 | 77.83 | 69.66 |
| Reads length | 150 bp | 26.11 kb | 150 bp |
| Coverage depth (×) | 84.05 | 74.12 | 66.34 |

**Table S2. Quality values of the assembled contig-level genome of *E. vivipara*.**

| Type | Contig length (Mb) |
| --- | --- |
| N10 | 6.86 |
| N20 | 4.67 |
| N30 | 3.49 |
| N40 | 2.67 |
| N50 | 2.17 |
| N60 | 1.73 |
| N70 | 1.22 |
| N80 | 0.75 |
| N90 | 0.35 |
| Contig Number | 1822 |
| Max length | 16.30 |
| Average length | 0.53 |
| Total length | 964.84 |

**Table S3. The completeness evaluation of the contig-level *E. vivipara* genome based on BUSCO database, mapping ratio of DNB short reads, and transcriptome data.**

| Genome features | Value |
| --- | --- |
| BUSCO value (%) | 94.23 |
| Single Copy (%) | 23.85 |
| Duplicated (%) | 70.38 |
| Fragment (%) | 1.90 |
| Mapping ratio of DNB-seq (%) | 95.80 |
| Mapping ratio of mRNA-seq (%) | 91.74 |
| Mapping ratio of Hi-C data (%) | 96.13 |

**Table S4. Detail of the ten long chromosomes constructed with Hi-C interaction intensity map.**

| Chromosomes | Length (Mb) | Gene number |
| --- | --- | --- |
| Chr1A | 116.72 | 4433 |
| Chr1B | 115.52 | 4409 |
| Chr2A | 86.38 | 3817 |
| Chr2B | 83.91 | 3736 |
| Chr3A | 98.39 | 4326 |
| Chr3B | 90.96 | 3867 |
| Chr4A | 78.19 | 3111 |
| Chr4B | 77.72 | 3100 |
| Chr5A | 93.93 | 3843 |
| Chr5B | 86.84 | 3644 |
| Unanchored | 36.68 | 483 |
| Total | 965.22 | 38769 |

**Table S5. The content of repeat sequence in *E. vivipara* chromosome-level genome.**

| Type | Number of elements | Length (bp) | Percentages of the genome (%) |
| --- | --- | --- | --- |
| DNA | 1581 | 532187 | 0.06 |
| CMC-EnSpm | 31545 | 8910278 | 0.92 |
| CMC-Transib | 542 | 65422 | 0.01 |
| Crypton-H | 739 | 168068 | 0.02 |
| DTA | 80911 | 19803101 | 2.05 |
| DTC | 66610 | 15425650 | 1.6 |
| DTH | 9280 | 1564589 | 0.16 |
| DTM | 129517 | 25473284 | 2.64 |
| DTT | 5690 | 1209459 | 0.13 |
| Helitron | 702644 | 132227175 | 13.70 |
| MULE-F | 98 | 25392 | 0 |
| MULE-MuDR | 10795 | 8381203 | 0.87 |
| Maverick | 1430 | 474618 | 0.05 |
| P | 45 | 8924 | 0 |
| PIF-Harbinger | 9287 | 7570227 | 0.78 |
| TcMar-Fot1 | 244 | 75128 | 0.01 |
| TcMar-Pogo | 207 | 109485 | 0.01 |
| TcMar-Tc1 | 426 | 71087 | 0.01 |
| hAT | 2478 | 713211 | 0.07 |
| hAT-Ac | 12691 | 5020145 | 0.52 |
| hAT-Tag1 | 2988 | 818267 | 0.08 |
| hAT-Tip100 | 3530 | 866930 | 0.09 |
| LINE |  |  | 0 |
| CR1 | 342 | 162409 | 0.02 |
| L1 | 15687 | 8549041 | 0.89 |
| L2 | 1380 | 495465 | 0.05 |
| R2-NeSL | 72 | 6377 | 0 |
| RTE-BovB | 4168 | 4423292 | 0.46 |
| unknown | 1189 | 783047 | 0.08 |
| LTR | 103 | 37605 | 0 |
| Cassandra | 29 | 1895 | 0 |
| Caulimovirus | 2202 | 6851564 | 0.71 |
| Copia | 83898 | 96376554 | 9.98 |
| ERV1 | 1051 | 397436 | 0.04 |
| Gypsy | 76819 | 119343964 | 12.36 |
| Ngaro | 3577 | 747767 | 0.08 |
| unknown | 71845 | 33573830 | 3.48 |
| MITE |  |  |  |
| DTA | 25313 | 4490758 | 0.47 |
| DTC | 1878 | 249267 | 0.03 |
| DTH | 5735 | 778348 | 0.08 |
| DTM | 29100 | 3042322 | 0.32 |
| DTT | 80 | 7695 | 0 |
| RC |  |  |  |
| Helitron | 3694 | 1660105 | 0.17 |
| Retroposon |  |  |  |
| SVA | 51 | 16700 | 0 |
| SINE |  |  |  |
| tRNA | 152 | 21489 | 0 |
| tRNA-Core | 3123 | 312355 | 0.03 |
| tRNA-RTE | 455 | 54843 | 0.01 |
| TIR |  |  |  |
| MuDR_Mutator | 306 | 111659 | 0.01 |
| PIF_Harbinger | 292 | 153244 | 0.02 |
| hAT | 40 | 18275 | 0 |
| Unknown | 332807 | 95268597 | 9.87 |
| Pararetrovirus | 220 | 183598 | 0.02 |
| Low_complexity | 28168 | 1341842 | 0.14 |
| Satellite | 1265 | 431989 | 0.04 |
| Simple_repeat | 142261 | 6659648 | 0.69 |
| rRNA | 490 | 120482 | 0.01 |
| snRNA | 79 | 16228 | 0 |
| Total | 1911149 | 616203520 | 63.84 |

**Table S6. Features of the assembly and protein-coding genes for the chromosome-level genome of *E. vivipara*.**

| Features for the genome of *E. vivipara* | Value |
| --- | --- |
| Contig N50 (Mb) | 2.17 |
| Scaffold N50 (Mb) | 90.96 |
| Busco (%) | 94.23 |
| Ploidy level | 4 |
| Number of protein-coding genes | 38769 |
| Number of transcripts | 61310 |
| Average protein-coding genes length (bp) | 4475.81 |
| Average transcript length (bp) | 4377.43 |
| Average coding sequence length (bp) | 1206.36 |

**Table S7.** **Function annotation of *E. vivipara* genes based on the five databases.**

| Databases | Gene number | Percentage (%) |
| --- | --- | --- |
| KEGG | 17135 | 44.20 |
| GO | 17011 | 43.88 |
| NR | 35048 | 90.40 |
| Swissprot | 29825 | 76.93 |
| Pfam | 28210 | 72.76 |
| Total | 35094 | 90.52 |

**Table S8. Comparison of gene families for the thirteen species in phylogenetic tree.**

| Species | Number of  genes | Number of gene  families | Number of  genes in gene  families | Number of unclustered  genes |
| --- | --- | --- | --- | --- |
| *C. esculentus* | 23606 | 13880 | 23400 | 206 |
| 1. *vivipara* | 38769 | 14555 | 34963 | 3806 |
| *C. scoparia* | 25799 | 16168 | 24206 | 1593 |
| *C. cristatella* | 26500 | 16260 | 24952 | 1548 |
| *C. littledalei* | 22441 | 13905 | 21582 | 859 |
| *R. pubera* | 91363 | 14837 | 79188 | 12175 |
| *R. breviuscula* | 24354 | 13679 | 22320 | 2034 |
| *R. tenuis* | 23215 | 13389 | 22176 | 1039 |
| *J. inflexus* | 25422 | 15176 | 23284 | 2138 |
| *J. effusus* | 25967 | 15466 | 23695 | 2272 |
| 1. *mays* | 39756 | 16043 | 35883 | 3873 |
| 1. *sativa* | 42580 | 15982 | 40734 | 1846 |
| 1. *thaliana* | 27416 | 12700 | 24728 | 2688 |

**Table S9. Gene ontology (GO) enrichment of genes in expanded (A), significant expanded (B), contracted (C), significant contracted (D), and unique (E) gene families of *E. vivipara* genome.** BP, biological process.

| (A) | | | | | | | | | | | |
| --- | --- | --- | --- | --- | --- | --- | --- | --- | --- | --- | --- |
| GO term | | Ontology | | | Description | | P-value | | Q-value | | |
| GO:0015979 | | BP | | | photosynthesis | | 6.70E-10 | | 3.84E-06 | | |
| GO:0019684 | | BP | | | photosynthesis, light reaction | | 1.36E-09 | | 3.84E-06 | | |
| GO:0044085 | | BP | | | cellular component biogenesis | | 1.73E-09 | | 3.84E-06 | | |
| GO:0006091 | | BP | | | generation of precursor metabo-lites and energy | | 2.19E-08 | | 3.65E-05 | | |
| GO:0071840 | | BP | | | cellular component organization or biogenesis | | 4.98E-08 | | 6.64E-05 | | |
| GO:0010639 | | BP | | | negative regulation of organelle organization | | 2.11E-07 | | 2.34E-04 | | |
| GO:0006996 | | BP | | | organelle organization | | 2.80E-07 | | 2.36E-04 | | |
| GO:0043933 | | BP | | | macromolecular complex subunit organization | | 2.84E-07 | | 2.36E-04 | | |
| GO:0019363 | | BP | | | pyridine nucleotide biosynthetic process | | 6.09E-07 | | 4.51E-04 | | |
| GO:0019359 | | BP | | | nicotinamide nucleotide biosynthetic process | | 1.12E-06 | | 7.46E-04 | | |
| GO:2001251 | | BP | | | negative regulation of chromosome organization | | 2.30E-06 | | 1.28E-03 | | |
| GO:0006793 | | BP | | | phosphorus metabolic process | | 2.30E-06 | | 1.28E-03 | | |
| GO:0009765 | | BP | | | photosynthesis, light harvesting | | 2.79E-06 | | 1.43E-03 | | |
| GO:0016071 | | | BP | | mRNA metabolic process | | 3.16E-06 | | 1.50E-03 | | |
| GO:0006796 | | BP | | | phosphate-containing compound metabolic process | | 3.77E-06 | | 1.59E-03 | | |
| GO:0022613 | | BP | | | ribonucleoprotein complex biogenesis | | 3.88E-06 | | 1.59E-03 | | |
| GO:0072525 | | BP | | | pyridine-containing compound biosynthetic process | | 4.07E-06 | | 1.59E-03 | | |
| GO:0022607 | | BP | | | cellular component assembly | | 4.32E-06 | | 1.60E-03 | | |
| GO:0009451 | | BP | | | RNA modification | | 4.88E-06 | | 1.71E-03 | | |
| GO:0022411 | | BP | | | cellular component disassembly | | 5.44E-06 | | 1.81E-03 | | |
| GO:0009767 | | BP | | | photosynthetic electron transport chain | | 1.26E-05 | | 3.71E-03 | | |
| GO:0051129 | | BP | | | negative regulation of cellular component organization | | 1.27E-05 | | 3.71E-03 | | |
| GO:0043412 | | BP | | | macromolecule modification | | 1.30E-05 | | 3.71E-03 | | |
| GO:0000377 | | BP | | | RNA splicing, via transesterification reactions with bulged adenosine as nucleophile | | 1.34E-05 | | 3.71E-03 | | |
| GO:0009744 | | BP | | | response to sucrose | | 1.45E-05 | | 3.71E-03 | | |
| (B) | | | | | | | | | | | |
| GO term | | Ontology | | | Description | | P-value | | Q-value | | |
| GO:0006796 | | BP | | | phosphate-containing compound metabolic process | | 1.25E-11 | | 3.07E-08 | | |
| GO:0006793 | | BP | | | phosphorus metabolic process | | 1.29E-11 | | 3.07E-08 | | |
| GO:0016310 | | BP | | | phosphorylation | | 3.65E-11 | | 5.80E-08 | | |
| GO:0000281 | | BP | | | mitotic cytokinesis | | 6.11E-09 | | 6.02E-06 | | |
| GO:0006468 | | BP | | | protein phosphorylation | | 6.31E-09 | | 6.02E-06 | | |
| GO:0048511 | | BP | | | rhythmic process | | 8.04E-09 | | 6.39E-06 | | |
| GO:0007623 | | BP | | | circadian rhythm | | 1.61E-08 | | 1.10E-05 | | |
| GO:0030004 | | BP | | | cellular monovalent inorganic cation homeostasis | | 3.46E-08 | | 2.07E-05 | | |
| GO:0009920 | | BP | | | cell plate formation involved in plant-type cell wall biogenesis | | 4.17E-08 | | 2.21E-05 | | |
| GO:0061640 | | BP | | | cytoskeleton-dependent cytokinesis | | 1.18E-07 | | 5.65E-05 | | |
| GO:0019359 | | BP | | | nicotinamide nucleotide biosynthetic process | | 3.61E-07 | | 1.56E-04 | | |
| GO:0030641 | | BP | | | regulation of cellular pH | | 4.24E-07 | | 1.56E-04 | | |
| GO:0051453 | | BP | | | regulation of intracellular pH | | 4.24E-07 | | 1.56E-04 | | |
| GO:0035556 | | BP | | | intracellular signal transduction | | 5.22E-07 | | 1.78E-04 | | |
| GO:0032506 | | BP | | | cytokinetic process | | 6.11E-07 | | 1.82E-04 | | |
| GO:1902410 | | BP | | | mitotic cytokinetic process | | 6.11E-07 | | 1.82E-04 | | |
| GO:0000910 | | BP | | | cytokinesis | | 7.32E-07 | | 2.03E-04 | | |
| GO:0006535 | | BP | | | cysteine biosynthetic process from serine | | 7.67E-07 | | 2.03E-04 | | |
| GO:0046496 | | BP | | | nicotinamide nucleotide metabolic process | | 9.54E-07 | | 2.31E-04 | | |
| GO:0000919 | | BP | | | cell plate assembly | | 9.71E-07 | | 2.31E-04 | | |
| GO:0019363 | | BP | | | pyridine nucleotide biosynthetic process | | 1.02E-06 | | 2.32E-04 | | |
| GO:0009072 | | BP | | | aromatic amino acid family metabolic process | | 1.46E-06 | | 3.16E-04 | | |
| GO:0000911 | | BP | | | cytokinesis by cell plate formation | | 1.71E-06 | | 3.55E-04 | | |
| GO:0019362 | | BP | | | pyridine nucleotide metabolic process | | 2.02E-06 | | 4.02E-04 | | |
| GO:1903959 | | BP | | | regulation of anion transmembrane transport | | 2.42E-06 | | 4.54E-04 | | |
| (C) | | | | | | | | | | | |
| GO term | Ontology | | | Description | | P-value | | | | Q-value | |
| GO:0060416 | BP | | | response to growth hormone | | 3.02E-17 | | | | 4.27E-14 | |
| GO:0048576 | BP | | | positive regulation of short-day photoperiodism, flowering | | 2.05E-14 | | | | 1.45E-11 | |
| GO:0009407 | BP | | | toxin catabolic process | | 6.48E-14 | | | | 2.29E-11 | |
| GO:0090487 | BP | | | secondary metabolite catabolic process | | 6.48E-14 | | | | 2.29E-11 | |
| GO:0080148 | BP | | | negative regulation of response to water deprivation | | 3.16E-13 | | | | 8.92E-11 | |
| GO:0006749 | BP | | | glutathione metabolic process | | 4.92E-12 | | | | 1.16E-09 | |
| GO:0009704 | BP | | | de-etiolation | | 7.61E-12 | | | | 1.34E-09 | |
| GO:0043434 | BP | | | response to peptide hormone | | 7.61E-12 | | | | 1.34E-09 | |
| GO:1901652 | BP | | | response to peptide | | 2.06E-11 | | | | 3.23E-09 | |
| GO:0048587 | BP | | | regulation of short-day photoperiodism, flowering | | 7.58E-11 | | | | 1.07E-08 | |
| GO:0009404 | BP | | | toxin metabolic process | | 1.09E-10 | | | | 1.40E-08 | |
| GO:0048572 | BP | | | short-day photoperiodism | | 4.59E-10 | | | | 4.98E-08 | |
| GO:0048575 | BP | | | short-day photoperiodism, flowering | | 4.59E-10 | | | | 4.98E-08 | |
| GO:0009640 | BP | | | photomorphogenesis | | 5.10E-09 | | | | 5.14E-07 | |
| GO:0006575 | BP | | | cellular modified amino acid metabolic process | | 1.74E-08 | | | | 1.64E-06 | |
| GO:0009791 | BP | | | post-embryonic development | | 3.02E-08 | | | | 2.66E-06 | |
| GO:0010470 | BP | | | regulation of gastrulation | | 4.76E-08 | | | | 3.74E-06 | |
| GO:1903224 | BP | | | regulation of endodermal cell differentiation | | 4.76E-08 | | | | 3.74E-06 | |
| GO:0048731 | BP | | | system development | | 7.59E-08 | | | | 5.64E-06 | |
| GO:0044248 | BP | | | cellular catabolic process | | 2.47E-07 | | | | 1.74E-05 | |
| GO:0009056 | BP | | | catabolic process | | 3.04E-07 | | | | 2.04E-05 | |
| GO:0022622 | BP | | | root system development | | 4.26E-07 | | | | 2.61E-05 | |
| GO:0048364 | BP | | | root development | | 4.26E-07 | | | | 2.61E-05 | |
| GO:0051603 | BP | | | proteolysis involved in cellular protein catabolic process | | 4.71E-07 | | | | 2.65E-05 | |
| GO:0017062 | BP | | | respiratory chain complex III assembly | | 5.06E-07 | | | | 2.65E-05 | |
| (D) | | | | | | | | | | | |
| GO term | Ontology | | | Description | | P-value | | | | Q-value | |
| GO:0048731 | BP | | | system development | | 8.15E-09 | | | | 3.90E-06 | |
| GO:0010431 | BP | | | seed maturation | | 6.26E-08 | | | | 9.90E-06 | |
| GO:0071695 | BP | | | anatomical structure maturation | | 8.67E-08 | | | | 9.90E-06 | |
| GO:0048608 | BP | | | reproductive structure development | | 1.06E-07 | | | | 9.90E-06 | |
| GO:0061458 | BP | | | reproductive system development | | 1.06E-07 | | | | 9.90E-06 | |
| GO:0009791 | BP | | | post-embryonic development | | 1.24E-07 | | | | 9.90E-06 | |
| GO:0021700 | BP | | | developmental maturation | | 3.97E-07 | | | | 2.71E-05 | |
| GO:0003006 | BP | | | developmental process involved in reproduction | | 9.83E-07 | | | | 5.68E-05 | |
| GO:0007275 | BP | | | multicellular organism development | | 1.07E-06 | | | | 5.68E-05 | |
| GO:0044702 | BP | | | single organism reproductive process | | 1.62E-06 | | | | 7.76E-05 | |
| GO:0044707 | BP | | | single-multicellular organism process | | 2.80E-06 | | | | 1.22E-04 | |
| GO:0032501 | BP | | | multicellular organismal process | | 6.52E-06 | | | | 2.53E-04 | |
| GO:0048856 | BP | | | anatomical structure development | | 6.87E-06 | | | | 2.53E-04 | |
| GO:0022414 | BP | | | reproductive process | | 7.67E-06 | | | | 2.61E-04 | |
| GO:0000003 | BP | | | reproduction | | 8.19E-06 | | | | 2.61E-04 | |
| GO:0009812 | BP | | | flavonoid metabolic process | | 1.08E-05 | | | | 3.23E-04 | |
| GO:0044767 | BP | | | single-organism developmental process | | 1.59E-05 | | | | 4.40E-04 | |
| GO:0009908 | BP | | | flower development | | 1.66E-05 | | | | 4.40E-04 | |
| GO:0090567 | BP | | | reproductive shoot system development | | 2.24E-05 | | | | 5.64E-04 | |
| GO:0048658 | BP | | | anther wall tapetum development | | 2.43E-05 | | | | 5.80E-04 | |
| GO:0032502 | BP | | | developmental process | | 2.64E-05 | | | | 6.01E-04 | |
| GO:0010623 | BP | | | developmental programmed cell death | | 4.07E-05 | | | | 8.84E-04 | |
| GO:0048609 | BP | | | multicellular organismal reproductive process | | 6.38E-05 | | | | 1.33E-04 | |
| GO:0009735 | BP | | | response to cytokinin | | 7.50E-05 | | | | 1.49E-04 | |
| GO:0032504 | BP | | | multicellular organism reproduction | | 1.04E-04 | | | | 1.99E-03 | |
| (E) | | | | | | | | | | | |
| GO term | Ontology | | | Description | | | | P-value | | | Q-value |
| GO:0034724 | BP | | | DNA replication-independent nucleosome organization | | | | 7.59E-08 | | | 8.17E-05 |
| GO:0006305 | BP | | | DNA alkylation | | | | 1.06E-07 | | | 8.17E-05 |
| GO:0006306 | BP | | | DNA methylation | | | | 1.06E-07 | | | 8.17E-05 |
| GO:0044728 | BP | | | DNA methylation or demethylation | | | | 1.32E-07 | | | 8.17E-05 |
| GO:1902466 | BP | | | positive regulation of histone H3K27 trimethylation | | | | 2.51E-07 | | | 1.24E-04 |
| GO:0006304 | BP | | | DNA modification | | | | 6.08E-07 | | | 2.50E-04 |
| GO:1902464 | BP | | | regulation of histone H3K27 trimethylation | | | | 1.44E-06 | | | 5.08E-04 |
| GO:0007549 | BP | | | dosage compensation | | | | 2.82E-06 | | | 5.35E-04 |
| GO:0009048 | BP | | | dosage compensation by inactivation of X chromosome | | | | 2.82E-06 | | | 5.35E-04 |
| GO:0090670 | BP | | | RNA localization to Cajal body | | | | 2.82E-06 | | | 5.35E-04 |
| GO:0090671 | BP | | | telomerase RNA localization to Cajal body | | | | 2.82E-06 | | | 5.35E-04 |
| GO:0090672 | BP | | | telomerase RNA localization | | | | 2.82E-06 | | | 5.35E-04 |
| GO:1904872 | BP | | | regulation of telomerase RNA localization to Cajal body | | | | 2.82E-06 | | | 5.35E-04 |
| GO:0098532 | BP | | | histone H3K27 trimethylation | | | | 5.06E-06 | | | 8.80E-04 |
| GO:0080188 | BP | | | RNA-directed DNA methylation | | | | 5.35E-06 | | | 8.80E-04 |
| GO:0032968 | BP | | | positive regulation of transcription elongation from RNA polymerase II promoter | | | | 7.98E-06 | | | 1.20E-03 |
| GO:0032844 | BP | | | regulation of homeostatic process | | | | 8.24E-06 | | | 1.20E-03 |
| GO:0016999 | BP | | | antibiotic metabolic process | | | | 1.16E-05 | | | 1.33E-03 |
| GO:0032204 | BP | | | regulation of telomere maintenance | | | | 1.18E-05 | | | 1.33E-03 |
| GO:0016075 | BP | | | rRNA catabolic process | | | | 1.28E-05 | | | 1.33E-03 |
| GO:0032786 | BP | | | positive regulation of DNA-templated transcription, elongation | | | | 1.28E-05 | | | 1.33E-03 |
| GO:0071030 | BP | | | nuclear mRNA surveillance of spliceosomal pre-mRNA splicing | | | | 1.35E-05 | | | 1.33E-03 |
| GO:0071034 | BP | | | CUT catabolic process | | | | 1.35E-05 | | | 1.33E-03 |
| GO:0071043 | BP | | | CUT metabolic process | | | | 1.35E-05 | | | 1.33E-03 |
| GO:0071048 | BP | | | nuclear retention of unspliced pre-mRNA at the site of transcription | | | | 1.35E-05 | | | 1.33E-03 |

**Table S10. Summary of mRNA-seq data and numbers of expressed genes in each replicate.**

| Types of culms | Data Volume  (Gb) | Mapping ratio  (%) | Number of  expressed genes |
| --- | --- | --- | --- |
| 0 d-1 | 7.82 | 91.45 | 27077 |
| 0 d-2 | 8.16 | 88.68 | 27035 |
| 0 d-3 | 8.32 | 86.62 | 26731 |
| 1 d-1 | 7.64 | 90.37 | 23904 |
| 1 d-2 | 8.22 | 89.68 | 24288 |
| 1 d-3 | 8.98 | 93.61 | 24540 |
| 9 d-1 | 6.89 | 98.65 | 25228 |
| 9 d-2 | 3.30 | 98.64 | 25058 |
| 9 d-3 | 3.03 | 98.55 | 26182 |
| 13 d-1 | 6.59 | 89.32 | 26318 |
| 13 d-2 | 6.82 | 89.78 | 25853 |
| 13 d-3 | 9.64 | 85.02 | 25965 |
| 30 d-1 | 7.90 | 92.50 | 28534 |
| 30 d-2 | 7.60 | 95.74 | 27961 |
| 30 d-3 | 7.39 | 92.60 | 27272 |
| 60 d-1 | 7.94 | 94.71 | 22915 |
| 60 d-2 | 6.46 | 93.53 | 21549 |
| 60 d-3 | 6.73 | 92.75 | 21697 |
| Total | 129.43 | 91.75 | 31939 |

**Table S11. List of primer sequences for selected genes used to perform the RT-qPCR experiment.**

| Primer names | Primer Sequence | Gene Functions | DEGs |
| --- | --- | --- | --- |
| *ELviv08G0332760*-F | ACGAGCAGGCTGAGTTGATT | C4 core pathway  (NAD-ME) | Yes |
| *ELviv08G0332760*-R | TTGTTGGCTGCTGTGCTAGA |  |  |
| *ELviv03G0151860*-F | TTCTCCAGTCTTTGGCTCGG | C4 core pathway  (AlaAT) | Yes |
| *ELviv03G0151860*-R | GATGCGCGGGAAGAGATACA |  |  |
| *ELviv10G0051240*-F | CCCTTGCCCTTTATATTCCCCT | C4 core pathway  (NADP-ME) | Yes |
| *ELviv10G0051240*-R | TGGTTCTAGTTCAAGTCGCTGT |  |  |
| *ELviv05G0212290*-F | AGTTCCTTTGGCTGTCGAGG | Response to flooding | Yes |
| *ELviv05G0212290*-R | CCACGCCACCCATCTAATGA |  |  |
| *ELviv04G0174130*-F | TTGCGCCATAGACCACTTGT | Response to light stimulus | No |
| *ELviv04G0174130*-R | GAACCAACAAGCGAAGCACC |  |  |
| *ELviv04G0190120*-F | GGTGAAGTGTTGACAGCCAG | Photorespiration | Yes |
| *ELviv04G0190120*-R | CTCGCTGTACCAGTGTTGCT |  |  |
| *ELviv06G0275750*-F | AGCAAAGACCGTGCAAATGG | Photosynthesis and chloroplast organization | Yes |
| *ELviv06G0275750*-R | TTCTTCTTGGGGAGCACGAC |  |  |
| *ELviv10G0058570*-F | AAACCAAGCTCTCGACCGAA | Photosynthesis and chloroplast organization | Yes |
| *ELviv10G0058570*-R | GGCAGTGGAGCAAGAAGAGA |  |  |
| *ELviv01G0032920*-F | AAGAATGGAATGCAGCGCAA | Photosynthesis and chloroplast organization | Yes |
| *ELviv01G0032920*-R | TGAGCAGCGCGAAGATTGTA |  |  |
| *ELviv10G0065580*-F | TTCTACCACGCTCCCCATTC | Photosynthesis and chloroplast organization | No |
| *ELviv10G0065580*-R | AGACGGAGAGGCACCATTTG |  |  |
| *ELviv09G0355300*-F | TAGAGTTACCGATGGCGCAG | Regulation of flower development | Yes |
| *ELviv09G0355300*-R | ACGCTTGCACAATTTTCTCCC |  |  |
| *ELviv04G0163450*-F | CCGGAAGAAAACTTGTGGGC | Actin | Yes |
| *ELviv04G0163450*-R | CATCGGCTTTTGCCTCATCG |  |  |

**Table S12.** **GO enrichment of genes in 2,602 up-DEGs (A) and 1,095 down-DEGs (B).** BP, biological process. MF, molecular function.

| (A) | | | | | | |
| --- | --- | --- | --- | --- | --- | --- |
| GO term | Ontology | Description | | P-value | | Q-value |
| GO:0006811 | BP | ion transport | | 4.93E-27 | | 1.47E-23 |
| GO:0055085 | BP | transmembrane transport | | 2.90E-26 | | 4.33E-23 |
| GO:0034220 | BP | ion transmembrane transport | | 6.90E-22 | | 6.85E-19 |
| GO:0001101 | BP | response to acid chemical | | 6.07E-18 | | 4.52E-15 |
| GO:1901700 | BP | response to oxygen-containing compound | | 1.09E-17 | | 6.47E-15 |
| GO:0006820 | BP | anion transport | | 2.62E-17 | | 1.30E-14 |
| GO:0015698 | BP | inorganic anion transport | | 3.40E-17 | | 1.45E-14 |
| GO:0010035 | BP | response to inorganic substance | | 2.81E-16 | | 1.05E-13 |
| GO:0042221 | BP | response to chemical | | 1.97E-14 | | 6.50E-12 |
| GO:0009415 | BP | response to water | | 1.39E-13 | | 4.14E-11 |
| GO:0098656 | BP | anion transmembrane transport | | 1.92E-13 | | 5.17E-11 |
| GO:0009414 | BP | response to water deprivation | | 2.09E-13 | | 5.17E-11 |
| GO:0010167 | BP | response to nitrate | | 2.48E-11 | | 5.69E-09 |
| GO:0055114 | BP | oxidation-reduction process | | 3.20E-11 | | 6.82E-09 |
| GO:0006833 | BP | water transport | | 5.47E-11 | | 1.02E-08 |
| GO:0042044 | BP | fluid transport | | 5.47E-11 | | 1.02E-08 |
| GO:0098660 | BP | inorganic ion transmembrane transport | | 1.35E-10 | | 2.36E-08 |
| GO:0009624 | BP | response to nematode | | 9.01E-10 | | 1.49E-07 |
| GO:0006970 | BP | response to osmotic stress | | 9.69E-10 | | 1.52E-07 |
| GO:0042445 | BP | hormone metabolic process | | 1.50E-09 | | 2.24E-07 |
| GO:0042493 | BP | response to drug | | 3.10E-09 | | 4.22E-07 |
| GO:0044281 | BP | small molecule metabolic process | | 3.12E-09 | | 4.22E-07 |
| GO:0009737 | BP | response to abscisic acid | | 4.15E-09 | | 5.24E-07 |
| GO:0044765 | BP | single-organism transport | | 4.22E-09 | | 5.24E-07 |
| GO:1901657 | BP | glycosyl compound metabolic process | | 4.85E-09 | | 5.77E-07 |
| GO:0022857 | MF | transmembrane transporter activity | | 2.06E-27 | | 1.94E-24 |
| GO:0005215 | MF | transporter activity | | 2.17E-22 | | 1.02E-19 |
| GO:0022891 | MF | substrate-specific transmembrane transporter activity | | 6.28E-21 | | 1.97E-18 |
| GO:0015103 | MF | inorganic anion transmembrane transporter activity | | 1.95E-20 | | 2.62E-18 |
| GO:0015114 | MF | phosphate ion transmembrane transporter activity | | 1.95E-20 | | 2.62E-18 |
| GO:0015318 | MF | inorganic solute uptake transmembrane transporter activity | | 1.95E-20 | | 2.62E-18 |
| GO:0015563 | MF | uptake transmembrane transporter activity | | 1.95E-20 | | 2.62E-18 |
| GO:0008509 | MF | anion transmembrane transporter activity | | 1.81E-19 | | 2.14E-17 |
| GO:0015075 | MF | ion transmembrane transporter activity | | 3.60E-19 | | 3.77E-17 |
| GO:0022892 | MF | substrate-specific transporter activity | | 1.54E-17 | | 1.45E-15 |
| GO:0022804 | MF | active transmembrane transporter activity | | 4.93E-13 | | 4.23E-11 |
| GO:0005372 | MF | water transmembrane transporter activity | | 3.91E-12 | | 2.84E-10 |
| GO:0015250 | MF | water channel activity | | 3.91E-12 | | 2.84E-10 |
| GO:0022838 | MF | substrate-specific channel activity | | 3.21E-10 | | 2.16E-08 |
| GO:0015267 | MF | channel activity | | 4.97E-10 | | 2.93E-08 |
| GO:0022803 | MF | passive transmembrane transporter activity | | 4.97E-10 | | 2.93E-08 |
| GO:0015291 | MF | secondary active transmembrane transporter activity | | 8.81E-10 | | 4.89E-08 |
| GO:0015292 | MF | uniporter activity | | 2.84E-07 | | 1.49E-05 |
| GO:0016491 | MF | oxidoreductase activity | | 1.13E-06 | | 5.62E-05 |
| GO:0004497 | MF | monooxygenase activity | | 1.68E-06 | | 7.94E-05 |
| GO:0016615 | MF | malate dehydrogenase activity | | 2.00E-06 | | 8.97E-05 |
| GO:0004553 | MF | hydrolase activity, hydrolyzing O-glycosyl compounds | | 2.17E-06 | | 9.08E-05 |
| GO:0004470 | MF | malic enzyme activity | | 2.22E-06 | | 9.08E-05 |
| GO:0008324 | MF | cation transmembrane transporter activity | | 2.32E-06 | | 9.10E-05 |
| GO:0022890 | MF | inorganic cation transmembrane transporter activity | | 2.98E-06 | | 1.12E-04 |
| (B) | | | | | | |
| GO term | Ontology | Description | P-value | | Q-value | |
| GO:0048581 | BP | negative regulation of post-embryonic development | 1.30E-12 | | 2.56E-09 | |
| GO:0009910 | BP | negative regulation of flower development | 1.65E-10 | | 1.23E-07 | |
| GO:0051093 | BP | negative regulation of developmental process | 1.86E-10 | | 1.23E-07 | |
| GO:0048580 | BP | regulation of post-embryonic development | 4.36E-10 | | 2.04E-07 | |
| GO:0050896 | BP | response to stimulus | 5.16E-10 | | 2.04E-07 | |
| GO:0051241 | BP | negative regulation of multicellular organismal process | 1.88E-09 | | 6.12E-07 | |
| GO:2000242 | BP | negative regulation of reproductive process | 2.17E-09 | | 6.12E-07 | |
| GO:2000026 | BP | regulation of multicellular organismal development | 4.19E-09 | | 9.64E-07 | |
| GO:0009628 | BP | response to abiotic stimulus | 4.39E-09 | | 9.64E-07 | |
| GO:0051239 | BP | regulation of multicellular organismal process | 5.34E-09 | | 1.05E-06 | |
| GO:0050793 | BP | regulation of developmental process | 8.21E-09 | | 1.48E-06 | |
| GO:0009909 | BP | regulation of flower development | 1.04E-08 | | 1.71E-06 | |
| GO:0042221 | BP | response to chemical | 1.47E-08 | | 2.24E-06 | |
| GO:0007623 | BP | circadian rhythm | 8.73E-08 | | 1.23E-05 | |
| GO:0048511 | BP | rhythmic process | 1.17E-07 | | 1.54E-05 | |
| GO:0042753 | BP | positive regulation of circadian rhythm | 4.09E-07 | | 5.05E-05 | |
| GO:0010345 | BP | suberin biosynthetic process | 5.08E-07 | | 5.91E-05 | |
| GO:0009698 | BP | phenylpropanoid metabolic process | 9.13E-07 | | 1.00E-04 | |
| GO:0044550 | BP | secondary metabolite biosynthetic process | 1.19E-06 | | 1.24E-04 | |
| GO:0019748 | BP | secondary metabolic process | 1.80E-06 | | 1.78E-04 | |
| GO:0080040 | BP | positive regulation of cellular response to phosphate starvation | 2.09E-06 | | 1.96E-04 | |
| GO:0048831 | BP | regulation of shoot system development | 3.52E-06 | | 3.16E-04 | |
| GO:0009699 | BP | phenylpropanoid biosynthetic process | 3.71E-06 | | 3.19E-04 | |
| GO:0090567 | BP | reproductive shoot system development | 6.70E-06 | | 5.42E-04 | |
| GO:0009908 | BP | flower development | 6.91E-06 | | 5.42E-04 | |
| GO:0001071 | MF | nucleic acid binding transcription factor activity | 1.07E-08 | | 3.25E-06 | |
| GO:0003700 | MF | transcription factor activity, sequence-specific DNA binding | 1.07E-08 | | 3.25E-06 | |
| GO:0043565 | MF | sequence-specific DNA binding | 2.72E-05 | | 4.18E-03 | |
| GO:0051536 | MF | iron-sulfur cluster binding | 3.74E-05 | | 4.18E-03 | |
| GO:0051540 | MF | metal cluster binding | 3.74E-05 | | 4.18E-03 | |
| GO:0016491 | MF | oxidoreductase activity | 4.12E-05 | | 4.18E-03 | |
| GO:0052689 | MF | carboxylic ester hydrolase activity | 7.51E-05 | | 5.54E-03 | |
| GO:0000975 | MF | regulatory region DNA binding | 9.11E-05 | | 5.54E-03 | |
| GO:0001067 | MF | regulatory region nucleic acid binding | 9.11E-05 | | 5.54E-03 | |
| GO:0044212 | MF | transcription regulatory region DNA binding | 9.11E-05 | | 5.54E-03 | |
| GO:0004857 | MF | enzyme inhibitor activity | 1.00E-04 | | 5.54E-03 | |
| GO:0048037 | MF | cofactor binding | 1.23E-04 | | 6.24E-03 | |
| GO:0001134 | MF | transcription factor activity, transcription factor recruiting | 4.90E-04 | | 2.13E-03 | |
| GO:0001135 | MF | transcription factor activity, RNA polymerase II transcription factor recruiting | 4.90E-04 | | 2.13E-03 | |
| GO:0009922 | MF | fatty acid elongase activity | 5.84E-04 | | 2.37E-02 | |
| GO:0004719 | MF | protein-L-isoaspartate (D-aspartate) O-methyltransferase activity | 8.64E-04 | | 2.77E-02 | |
| GO:0015369 | MF | calcium: proton antiporter activity | 8.64E-04 | | 2.77E-02 | |
| GO:0036033 | MF | mediator complex binding | 8.64E-04 | | 2.77E-02 | |
| GO:0051139 | MF | metal ion:proton antiporter activity | 8.64E-04 | | 2.77E-02 | |
| GO:0001076 | MF | transcription factor activity, RNA polymerase II transcription factor binding | 1.14E-03 | | 3.48E-02 | |
| GO:0004551 | MF | nucleotide diphosphatase activity | 1.27E-03 | | 3.68E-02 | |
| GO:0051864 | MF | histone demethylase activity (H3-K36 specific) | 2.54E-03 | | 6.60E-02 | |
| GO:0080042 | MF | ADP-glucose pyrophosphohydrolase activity | 2.54E-03 | | 6.60E-02 | |
| GO:0009882 | MF | blue light photoreceptor activity | 2.60E-03 | | 6.60E-02 | |
| GO:0008964 | MF | phosphoenolpyruvate carboxylase activity | 3.50E-03 | | 7.99E-02 | |

**Table S13. List of gene involved in C_4_ core pathway used to compare the contribution of subgenome A and B in *E. vivipara*.**

| Gene family | Gene | Gene ID | Subgenome |
| --- | --- | --- | --- |
| CA | *EvCA4* | *ELviv09G0374850* | A |
|  | *EvCA10* | *ELviv10G0052560* | B |
| PEPC | *EvPEPC3* | *ELviv09G0373620* | A |
|  | *EvPEPC4* | *ELviv09G0373710* | A |
|  | *EvPEPC6* | *ELviv10G0053750* | B |
|  | *EvPEPC8* | *ELviv10G0053790* | B |
| PCK | *EvPCK1* | *ELviv09G0356720* | A |
|  | *EvPCK2* | *ELviv10G0071090* | B |
| PPCK | *EvPPCK2* | *ELviv07G0281100* | A |
|  | *EvPPCK3* | *ELviv07G0281110* | A |
|  | *EvPPCK4* | *ELviv07G0281140* | A |
|  | *EvPPCK6* | *ELviv08G0347990* | B |
|  | *EvPPCK7* | *ELviv08G0348010* | B |
|  | *EvPPCK8* | *ELviv08G0348020* | B |
| NAD-MDH | *EvNAD-MDH1* | *ELviv01G0012330* | A |
|  | *EvNAD-MDH3* | *ELviv09G0367290* | A |
|  | *EvNAD-MDH4* | *ELviv02G0107690* | B |
|  | *EvNAD-MDH6* | *ELviv08G0331440* | B |
|  | *EvNAD-MDH7* | *ELviv10G0060640* | B |
|  | *EvNAD-MDH8* | *ELviv10G0065270* | B |
|  | *EvNAD-MDH9* | *ELvivUnG0387580* | Contig |
| NAD-ME | *EvNAD-ME1* | *ELviv07G0297020* | A |
|  | *EvNAD-ME2* | *ELviv07G0286420* | A |
|  | *EvNAD-ME3* | *ELviv07G0286440* | A |
|  | *EvNAD-ME4* | *ELviv08G0332760* | B |
| PPDK | *EvPPDK3* | *ELviv07G0311280* | A |
|  | *EvPPDK6* | *ELviv08G0318870* | B |
| PPDKRP | *EvPPDKRP1* | *ELviv01G0042600* | A |
|  | *EvPPDKRP3* | *ELviv02G0081170* | B |
| AspAT | *EvAspAT1* | *ELviv10G0054050* | B |
|  | *EvAspAT2* | *ELviv10G0054070* | B |
| AlaAT | *EvAlaAT1* | *ELviv03G0151860* | A |
|  | *EvAlaAT3* | *ELviv04G0173470* | A |
|  | *EvAlaAT6* | *ELviv06G0267710* | B |
